# Additive Manufacture of Small-Scale Metamaterial Structures for Acoustic and Ultrasonic Applications

**DOI:** 10.3390/mi12060634

**Published:** 2021-05-29

**Authors:** Alicia Gardiner, Paul Daly, Roger Domingo-Roca, James F. C. Windmill, Andrew Feeney, Joseph C. Jackson-Camargo

**Affiliations:** 1 Centre for Ultrasonic Engineering, Department of Electronic & Electrical Engineering, University of Strathclyde, Glasgow G1 1XW, UK; paul.daly@strath.ac.uk (P.D.); roger.domingo-roca@strath.ac.uk (R.D.-R.); james.windmill@strath.ac.uk (J.F.C.W.); joseph.jackson@strath.ac.uk (J.C.J.-C.); 2 Centre for Medical and Industrial Ultrasonics, James Watt School of Engineering, University of Glasgow, Glasgow G12 8QQ, UK; Andrew.Feeney@glasgow.ac.uk

**Keywords:** acoustic metamaterials, additive manufacturing, acoustics, ultrasonics

## Abstract

Acoustic metamaterials are large-scale materials with small-scale structures. These structures allow for unusual interaction with propagating sound and endow the large-scale material with exceptional acoustic properties not found in normal materials. However, their multi-scale nature means that the manufacture of these materials is not trivial, often requiring micron-scale resolution over centimetre length scales. In this review, we bring together a variety of acoustic metamaterial designs and separately discuss ways to create them using the latest trends in additive manufacturing. We highlight the advantages and disadvantages of different techniques that act as barriers towards the development of realisable acoustic metamaterials for practical audio and ultrasonic applications and speculate on potential future developments.

## 1. Introduction

### 1.1. Overview

The progression of research concerning the acoustic, vibrational and mechanical behaviour of small-scale structures is closely linked to the status of microfabrication/3D printing technologies [1,2]. As these manufacturing methods become more developed, increasingly complex smaller-scale structures can be printed with finer detail and accuracy. Therefore, the potential exists for opening up significant opportunities to create high-performance devices for sound propagation and sensing across audio and ultrasonic frequency regimes. One such beneficiary of recent progress in microscale manufacturing is the field of acoustic metamaterials (AMM)—artificial materials (and devices built from those materials) that manipulate acoustic fields in a variety of ways [3]. Despite extensive research, AMMs have yet to be fully realized in commercial applications, with few examples available of their current deployment in industry (primarily based in sound control) [4,5,6]. This is in part due to the prohibitive and logistical restrictions in manufacturing these structures; as such, experimental prototypes of AMMs are typically 3D-printed to achieve the required level of precision and size [7]. Hence, they are rapidly prototyped but difficult to manufacture in bulk. Additive manufacturing technology with the ability to print microscopic structures is still advancing; and there are now several viable methods to realize small-scale metamaterial structures. In this review, the range of AMMs under current investigation are explored, demonstrating the mechanisms by which their fabrication can be achieved, and their potential for practical application in the future for both acoustic and ultrasonic applications. Microscale printing techniques are discussed at length, with reference to significant printed small-scale examples documented in the literature. In this review, small-scale refers to structures with a minimum feature size of a few millimetres or lower, although optimum small features can be within the micro or nanoscales.

### 1.2. Additive Manufacturing

Microscale printing has only recently been demonstrated to any significant scope [8,9,10], despite early additive manufacturing technology emerging in the 1980s [11,12]. While there were methods proposed and patented earlier, Charles Hull is generally credited with creating the first 3D printer. This system, patented in 1986 [13], used stereolithography (SLA) and reportedly required several months to fabricate its first print. Other printing methods followed in quick succession, including fused deposition modelling (FDM), selective laser sintering (SLS), and laminated object manufacturing (LOM), all of which were successfully patented by the end of the 1980s. Since then, additive manufacture technology has progressed exponentially, and by the 2000s had established itself as a popular, cost-effective option for academia and industry alike for rapid prototyping [14], and 3D printing is now popular even at home for amateur enthusiasts. Nowadays, additive printing methods can produce 3D structures with resolution reaching the nanoscale while maintaining a high print quality, for example, exhibiting no visible defects under an electron microscope [15]. The elimination of visible defects at these scales is a vital enabler for printing complex structures for acoustic and ultrasonic applications, devices for which precise control of geometry is required to tune dynamic properties. Key barriers to the manufacture of microstructures, and therefore AMMs, for use in industry are (i) the ability to mass-produce; (ii) the minimum printable feature size; and (iii) the ability to print multi-material structures. Addressing these challenges is imperative to fully realize the potential of AMMs across multiple applications, including underwater acoustics [16], medical imaging and NDT [17], acoustic cloaking [18] and sound control/attenuation [19].

### 1.3. Acoustic Metamaterials

AMMs have been studied extensively [3,20,21,22,23,24] and can be described as a structure composed of (often) periodically repeated acoustic elements that can manipulate sound waves to produce novel and interesting effects. For example, they can achieve a negative effective bulk modulus and negative effective density [3,21]. Unlike natural materials, AMMs derive their unique properties from their structure rather than their innate material composition. The size of their periodic resonant units, also called “meta-atoms”, are closely linked to the operational wavelength. There is substantial interest in creating AMM devices at sub-wavelength scales [20], as when the resonant units are significantly smaller than the wavelength (typically 1/10 the size) [24], the elements can behave as one effective continuous medium—waves do not see the granularity of the medium. Early forms of AMMs have dimensions similar to their operational wavelength [25], and their properties derive from scattering. As such, the upper wavelength limit, and corresponding largest possible meta-atom size, in the ultrasonic regime (i.e., >20 kHz in air) is 17.25 millimetres, demonstrating the need for the development of smaller-scale AMMs. Acoustic wavelengths, with air as the transmission medium, range from 17.25 millimetres (at 20 kHz) to 17.25 metres (at 20 Hz)—this poses an issue to commercializing audio-range technologies with wavelength equivalent sizing, due to their unfeasible dimensions. Devices operating in a fluid medium [16] or mechanical capacity [26] also present similar issues. AMMs using local resonances can be subwavelength and are thus more attractive and applicable. Developing 3D printing technology with finer resolutions is vital to miniaturize AMM designs to a practical size and enable their use in potential applications. To manufacture AMMs that can operate at higher ultrasonic frequencies, or sub-wavelength devices at audio frequencies, there is a demand for printing technologies with resolution in the micro (or even nano) scale.

A noteworthy area of AMM research is active structures, whose material properties and/or physical geometries can be changed by external stimuli. Parameters of particular interest are tuneable bulk modulus and effective density [27,28,29,30,31,32]. In the context of this review, an active AMM indicates an AMM system whose acoustic properties and/or geometric structure can be modified using an external interaction. This external input could involve an applied magnetic field [33], electric current, mechanical energy [34], temperature, chemical energy, fluid filling [35], hydration [36], or various other energy inputs/outputs [35]. Active AMMs can be broadly separated into non-Hermitian (involving acoustic gain) or externally biased (where there is no transfer of energy with acoustic waves) [22]. A common trend in active AMMs is to incorporate piezo-electric elements that allow tuning via an applied electric field [37,38]. In traditional (or passive) AMMs, the material properties cannot be altered after fabrication, and the operational bandwidth is fixed and often narrow. One limitation of this is their unsuitability for potentially transformative applications requiring a broader range of frequencies. However, AMM structures with a tuneable bandwidth present a potential opportunity to surpass this limitation. Active AMMs often incorporate many materials, due to their novel modulation systems; therefore, progressing multi-material 3D printing would greatly benefit the production of active AMMs.

### 1.4. Scope of Review

This review explores the variety of small-scale AMMs currently in development, along with the 3D printing methods that show potential to manufacture them. The outlook and potential applications for these AMMs are discussed critically, highlighting the barriers to overcome to enable widespread use of AMMs in industry, with recommendations for promising manufacturing methods to progress the field of microscale acoustic metamaterials.

## 2. Acoustic Metamaterials

### 2.1. Background

AMMs are artificial structures designed to achieve novel material properties that can manipulate acoustic waves in ways beyond the scope of natural materials. For example, AMMs have been documented with the following unusual features: negative bulk modulus; negative effective density; negative refractive index; and sub-wavelength diffraction/focusing [3,20]. While AMMs have yet to be implemented widely in industry, they show promise for use in several potential applications, such as those requiring sound attenuation [39] or acoustic signal processing [40], and widely-used applications including non-destructive testing [17], and acoustic and ultrasound imaging [23].

**Figure 1:**
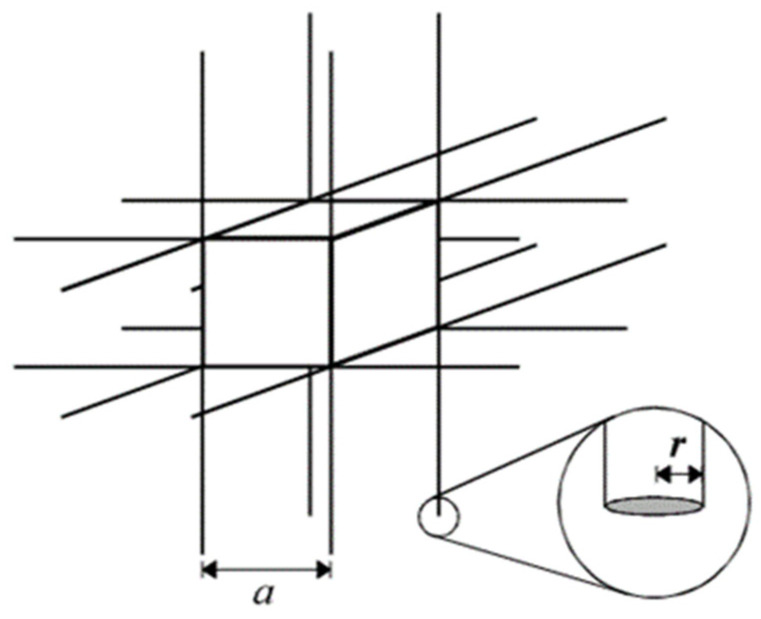
Pendry’s artificial microstructure design for manipulating low-frequency plasmons. Reprinted with permission from [43] (As follows: [J. B. Pendry et al., Physical Review Letters, Volume 76, Pages 4773–4776, 1996.], copyright (1996) by the American Physical Society).

Long before the term “metamaterials” was coined, there existed optical devices that demonstrated these extraordinary material properties. Arguably, the earliest conceived “metamaterial” was created in 1898 and was composed of jute fibres twisted in opposing directions [41]. This structure produced an optical twist in the plane of polarization of incident light waves, which is an effect not observed in any naturally-occurring materials. In 1968, Veselago theorized a material with a simultaneously negative dielectric constant and magnetic permeability [42], thereby beginning the research of metamaterials as a scientific field. These two properties are fundamental to the propagation of electromagnetic waves in matter, meaning this discovery offered new insight into the manipulation of waveform matter. Metamaterial research was largely theoretical until 1996, when Pendry proposed an artificial microstructure capable of manipulating very low frequency plasmons [43]. This design, as shown in Figure 1, consisted of a periodic lattice of thin metallic wires, arranged at increments close to the plasmon wavelength. The quantum band theory of solids used in Pendry’s design would also inspire the development of phononic crystals, an example of an early AMM. Given that optical phenomena can often find analogies in other wave phenomena such as acoustics, it is not surprising that by the late 1990s, acoustic band gaps in phononic crystals was demonstrated both in theory [44] and experimentally [45].

Just prior to Pendry’s artificial microstructure research, Helmholtz resonator arrays were among the first AMMs with potential applications discussed. In 1992, Sugimoto proposed using Helmholtz resonators embedded in a train tunnel to suppress shockwaves [46]. Phononic crystals, defined as a heterogeneous media composed of elastic inclusions in a periodic matrix, are capable of impeding acoustic wave propagation at specific frequencies [47]. By 2010, these artificial materials were researched and developed in one [48], two [49] and three [50] dimensions, allowing their design complexity to be tailored for the situational requirements. A key function of phononic crystals, which is the control of sound transmission spectra, has been explored for use in several potential applications, including sound filtering [1], wave guiding [51], radio-frequency communications [52] and acoustic sensors [53].

**Figure 2:**
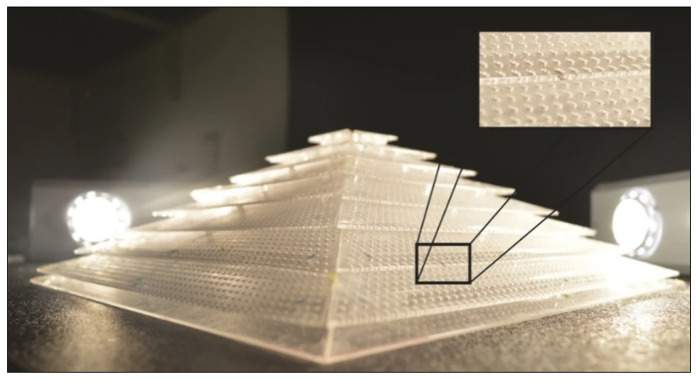
Omni-directional 3D acoustic cloak manufactured using 3D printing (cloak operational for acoustic waves at a frequency of 3 kHz) [57]. (Reprinted by permission from Springer Nature: [Nature Materials, ‘Three-dimensional broadband omnidirectional acoustic ground cloak’, L. Zigoneanu et al., 2014]).

A significant breakthrough in AMMs is the development of acoustic cloaks. Working similarly to an optical cloak, an acoustic cloak can shield an object from the surrounding acoustic field. A feasible 2D acoustic cloak prototype was first published in 2008 [54], building on a theoretical design proposed the previous year [55]. Following this, a 2D ultrasonic cloak for underwater operation was manufactured, consisting of a planar network of acoustic circuits machined into a circular aluminium plate [56]. This device successfully prevented a small test object from scattering oncoming pressure waves, demonstrating the feasibility of this technology to be used for sonar and other underwater applications. Omni-directional 3D cloaks are more complex to produce, but despite this, a working prototype was created in 2014 via 3D printing, and is shown in Figure 2 [57]. The device proved effective at reducing pressure-wave scattering in air at 3 kHz, and it is thus a viable option to meet the unrealized potential of acoustic cloaking. As additive manufacturing technology develops, this will allow 3D cloaks with fewer loss/scattering effects to be produced, and with functionality at broader frequency ranges. Current cloak designs are already showing an improvement in operational bandwidth [58]. This extraordinary invention has yet to be commercially available, but it is of interest for several airborne and underwater applications.

### 2.2. Types of AMM

AMMs have been separated into three distinct categories for the purposes of this review. These categories are (i) periodic/lattice metamaterials; (ii) coiled structures and metasurfaces; and (iii) active metamaterials. These classifications are not exclusive and there may be some overlap, with some AMM examples belonging to multiple categories.

#### 2.2.1. Periodic/Lattice Metamaterials

This type of AMM consists of periodic structures of repeating cells or meta-atoms. Phononic crystals describe a variety of lattice structures with fluid, elastic or combination inclusions in a different material matrix [21]. However, there is a distinct difference between these crystals and periodic AMMs, as the latter utilizes locally resonant unit-cells to manipulate acoustic waveforms. Phononic crystals possess lattice sizes on the same scale as their operational wavelength and function via scattering and interference of the incident waves globally through the lattice. In contrast, periodic AMMs use local resonance with minimal cross-talk between cells, meaning the wavelength and resonant frequencies are no longer reliant on the cell dimensions or global lattice parameters. It is the independent behaviour of these meta-atoms that allows sub-wavelength geometry to be achieved.

**Figure 3:**
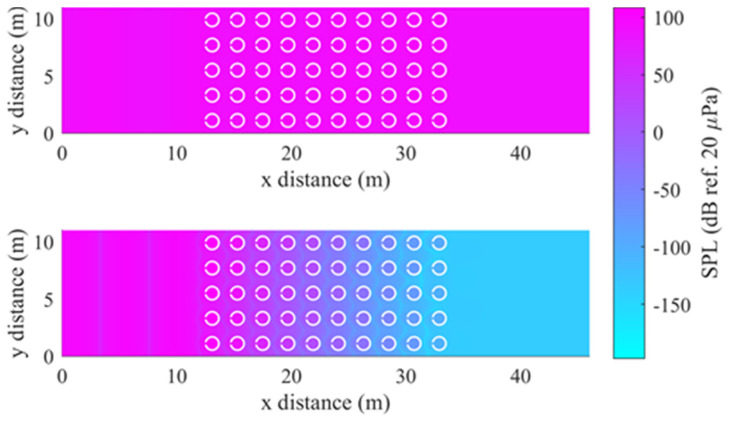
An FEA plot of sound pressure level of a Matryoshka locally resonant sonic crystal, modelled using COMSOL Multiphysics. Sound pressure levels are relatively consistent throughout when the incident wave has a frequency of 2 kHz (**top**) and lies outside the band gap but are attenuated considerably at 4 kHz (**bottom**) inside the gap.

Phononic crystals, despite their dimensional limitations, are still effective at sound attenuation applications. This is presented in a known phononic crystal design [59], which has been subsequently replicated in FEA to demonstrate its band gap properties. Figure 3 displays the resulting FEA sound pressure plot of the crystal at two key frequencies. The waves have frequencies of approximately 2 kHz and 4 kHz and lie without and within the band gap, respectively. In the former case, sound pressure levels (SPLs) remain relatively consistent throughout the whole domain, while in the latter, levels drop considerably through and after the crystal. The effect that causes this transmission loss is also apparent in the high pressures inside the resonating inclusions. In a phononic crystal (and other lattice types) the band gap is caused by Bragg scattering, whereby waves of a particular wavelength (determined by the lattice constant) are scattered from the inclusions and interfere destructively [60]. Acoustic meta-atoms that are smaller than the acoustic wavelength utilise gaps due to resonance effects [24]. If, in a metamaterial, both mechanisms are possible and the Bragg and resonance gaps can be coupled, the gap is called a hybridisation gap [61], which can be deeper than the gaps individually.

Periodic, locally-resonant AMMs can be produced in a variety of shapes and sizes, and from different materials, manufactured using both 3D printing and conventional fabrication techniques. The local resonances can be created using two different kinds of meta-atom: intrinsic and inertial [21,62]. Intrinsic AMMs consist of a single inclusion (with one bulk material) per unit-cell, with the phase speed of the inclusion much slower than that of the surrounding fluid matrix. Intrinsic AMMs are usually simpler, both in design and ability to manufacture without 3D printing. These materials were investigated in early AMM research [63,64,65,66], and as manufacturing methods became more sophisticated, this allowed the production of more complex periodic metamaterials.

**Figure 4:**
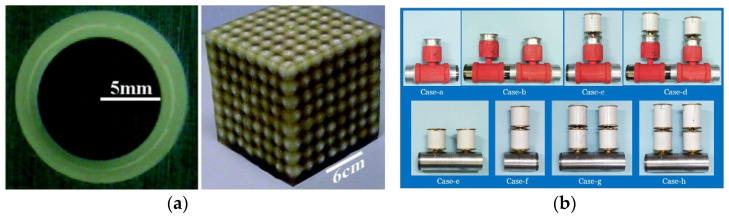
Examples of inertial meta-atom structures: with (**a**) showing a matrix of coated spherical inclusions (From [67]. Reprinted with permission from AAAS). Displayed in (**b**) are various configurations of Helmholtz resonator arrays [68] (Reprinted from Applied Acoustics, Volume 155, D.P. Jena, J. Dandsena, V.G. Jayakumari, ‘Demonstration of effective acoustic properties of different configurations of Helmholtz’, Pages 371-382, Copyright (2019), with permission from Elsevier).

**Figure 5:**
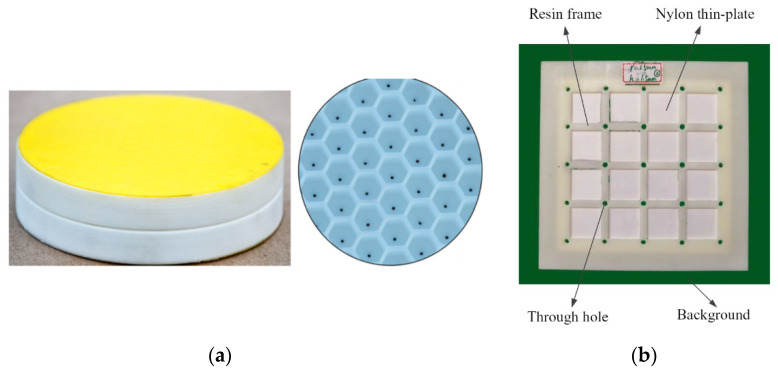
Examples of AMM devices incorporating membranes and perforated plates, where (**a**) displays a uniform metastructure of hexagonal unit-cells arranged in parallel and sealed by membranes at each open end (Reprinted from [71], with the permission of AIP Publishing). (**b**) shows a perforated thin-plate design with holes drilled at the cross points of the resin frame to introduce anti-resonant behaviour [73] (Republished with permission of IOP Publishing Ltd., from [Journal of Physics D: Applied Physics, Y. Xu, et al., Volume 52, Edition No. 40, 2019.]; permission conveyed through Copyright Clearance Center, Inc., Danvers, MA, USA).

Inertial meta-atoms operate as independent mechanical oscillators and can be mathematically modelled using a dynamic system of masses, springs and dampers. This allows accurate resonant behaviour prediction of increasingly complex, periodic metamaterials. Some inertial examples built upon existing intrinsic designs by adding coatings to spherical inclusions [67], while others involve arrays of Helmholtz resonators [68,69], periodic cavities within a material [70] and novel configurations of other mass–spring–damper (MSD) configurations, such as membranes [71,72], perforated plates [73] and internal masses [74]. Examples of these are shown in Figure 4 and Figure 5. As an extension to this type of AMM, holey-structured metamaterials have been used for acoustic imaging beyond the diffraction limit. An anisotropic AMM lens comprised of periodic air channels with different modulated diameters has achieved this through the coupling of evanescent waves [75].

Membrane-based acoustic metamaterials are capable of producing near complete reflection in a narrowband at low frequencies [76]. In their first configurations, a single mass was attached to an elastic rubber membrane that was held under tension and fixed to a relatively rigid frame [77]. Such a system has two resonant eigenmodes and, between them, an anti-resonant frequency where acceleration is out of phase with an incident pressure wave [78]. Consequently, membrane displacement and energy transmission are minimised. With an aim to increase the sound attenuating bandwidth, the effects of annular mass loadings and eccentric positioning have been investigated [79]. Some designs have incorporated arrays of membrane meta-units while others have stacked them in series [80]. The field remains active, with some emerging examples of 3D-printed membrane type metamaterials [81]. As the designs have become inexorably more complex, the manufacturing difficult has increased, and 3D printing is in our opinion both an enabler and a barrier to the development of more complex metamaterials.

#### 2.2.2. Coiled Structures and Metasurfaces

This sub-section is concerned with AMMs with chiral and coiled structures. These structures often, but not always, coincide with metasurfaces—defined as planar metamaterial and hence operating in 2D only. Given that these coiled structures are asymmetric, using two dimensions considerably simplifies the design. The coiled nature of these materials is inherently suitable for subwavelength devices, as propagating waves are made to travel much further than the global length of the structure.

**Figure 6:**
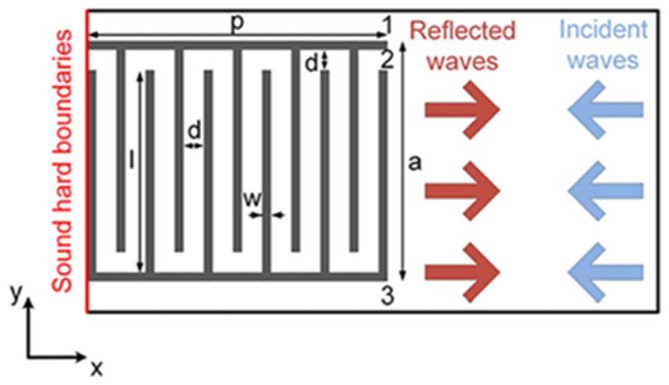
A 2D coiled structure acoustic metasurface [82].

An example of this type includes labyrinthian channels of air, and there is evidence that such a design proved capable of directing a phase shift in acoustic wavefronts [82]. Figure 6 shows an example of a 2D coiled design. In examples such as this, discrete increments of phase shift can be induced by modulating the width of the coiled structures. Another sub-wavelength metasurface employed Helmholtz resonators of various neck lengths to induce a phase change of up to 2π radians [83]. This structure is composed of eight periodic supercells of resonators with neck length tuned for phase changes in π/4 radian increments. Experimental testing of a (vat polymerization method) 3D-printed model proved the ability to steer an incoming acoustic wave, as well as converting it to a surface wave, thereby introducing negative reflection. An extraordinary attribute of this device is its deep sub-wavelength thickness, which was a factor of 1/30 smaller than the operational wavelength.

**Figure 7:**
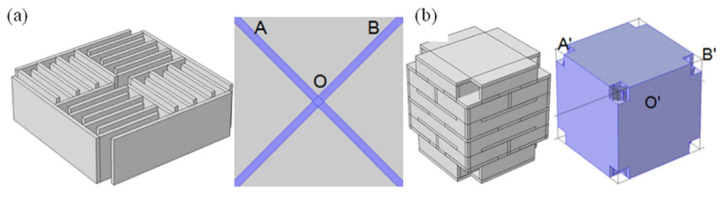
Coiled acoustic metamaterial structures in (**a**) 2D and (**b**) 3D, showing the equivalent cells in each case [85].

Another quality achievable by this AMM type is negative refraction [84]. This can provide superior wave focusing and attenuation at sub-wavelength scales. Figure 7 displays labyrinthine structures using negative refraction to attenuate sound, with designs implemented in both 2D and 3D [85]. Due to the coiled geometry and an impedance mismatch between the structure material and medium, negative effective density and bulk modulus present simultaneously at specific frequencies. Hence, the refractive index is negative, and superior sound dispersion is achieved within very narrow frequency bands.

**Figure 8:**
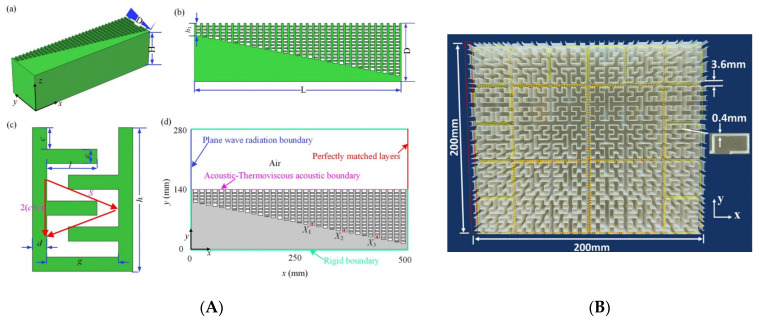
Broadband enhancement examples using acoustic metasurfaces. (**A**) displays a gradient coiled metamaterial with (**a**–**c**) presenting the design at various angles and cross-sections and (**d**) showing a diagram of the acoustic boundaries [86] (Republished with permission of IOP Publishing Ltd., from [Journal of Physics D: Applied Physics, T. Chen, et al., Volume 54, 2020.]; permission conveyed through Copyright Clearance Center, Inc.). (**B**) shows a 3D-printed fractal metamaterial design (Reprinted from [87], with the permission of AIP Publishing).

Coiled structures have also exhibited significant benefits in enhancing the broadband abilities of AMMs [86]. A gradient coiled metamaterial, printed with a vat polymerization 3D printing method, successfully magnified acoustic pressure amplitudes by approximately 80 times their original magnitude, compared to its non-coiled counterpart (straight channels with a gradient), which magnified pressures by a factor of 50. This gradient AMM is displayed in Figure 8. Additionally, propagation depth varies with frequency, where high frequency components are dissipated in shallower areas and low frequencies in deeper areas of the coiled structure. This acoustic phenomenon is called rainbow trapping, with the AMM structure acting a means of sensing and filtering different signals by their frequency. Hilbert fractals have also been considered for broadband sound absorption, specifically for low frequencies [87]. To illustrate, a fractal metasurface device is shown in Figure 8. By selectively arranging different-order fractal meta-atoms, this device achieved wide spectrum absorption of soundwaves between 225 Hz and 1175 Hz. This prototype was constructed with photosensitive resin (via 3D printing) and incorporated a wall thickness of 400 μm. As features in metasurface designs approach the microscale, 3D printing is increasingly used as the primary manufacturing method, allowing the complexity to be within structures more easily.

#### 2.2.3. Active Metamaterials

This category includes controlled, time-variant AMMs, meaning their material properties, physical structure or acoustic effects can be altered during use and are not constant variables. These materials can be modulated using a variety of external inputs and can function either with or without exchanging energy gain from the external acoustic field [22]. This branch of AMMs addresses some key issues hindering the use of AMMs in applications, because of their versatility in design and unprecedented tuneable properties.

**Figure 9:**
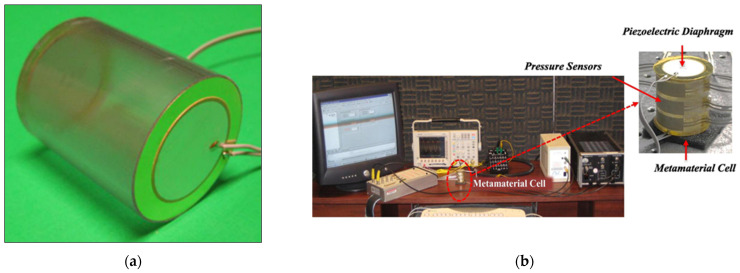
(**a**) Initial experimental prototype of an active AMM cell comprised of an acrylic cylinder with piezoelectric bimorphs sealing the ends. The volume of the cell is filled with water to act as the transmission medium. The piezoelectric membranes act as both actuators and sensing apparatus; hence they are connected via electrodes to a controller and amplifier (Reprinted from [38], with the permission of AIP Publishing). (**b**) Experimental set-up for an active AMM cell using piezoelectric membranes. A similar but more refined version of the experimental prototype in (**a**), with this example being more compact with additional embedded pressure sensors and closed-loop control [88] (Reprinted from Applied Acoustics, Volume 178, W. Akl and A. Baz., ‘Active control of the dynamic density of acoustic metamaterials’, Copyright (2021), with permission from Elsevier).

A common feedback method in active AMMs involves using piezoelectric material to convert acoustic pressure into electric current and vice versa. Initial active AMM designs consisted of rudimentary fluid cells separated by piezoelectric boundaries [30]. Figure 9a displays an experimental model of this initial active cell [38], which was tested and confirmed as a viable device for tuning the effective density. To achieve this, an electric charge is applied to the piezoelectric diaphragms, which varies the cell stiffness, and the active material is coupled with the fluid medium. The resulting outcome is a relatively broad tuneable effective density. This variation of active AMMs has been developed further in recent years, including implementing closed-loop control over the effective density of multi-cell systems [37] and demonstrating it experimentally [88]. This experimental meta-cell, shown in Figure 9b, achieved a wide range of effective density values (0.35–13 times that of the contained fluid medium). Another novel proposal, building on the aforementioned active AMM research, employs a gyroscopic AMM cell to control the magnitude and direction of propagating acoustic waves [89]. Developing this technology further shows promise for the future construction of practical acoustic cloaks [90] and high-performance tuneable acoustic filters [91].

**Figure 10:**
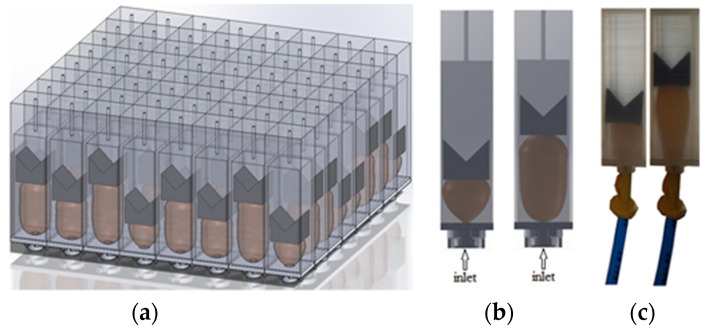
Tuneable Helmholtz resonator showing (**a**) a rendering of an experimental metamaterial design, composed of tuneable Helmholtz resonators that are actuated by inflatable balloons, (**b**) the variations of the plunger clearance depicted with the balloon inflated and deflated and (**c**) clearances as shown in the experimental prototype [92].

Other actuation methods for active AMMs include mechanical energy, temperature and applied magnetic fields. Helmholtz resonator-based devices can incorporate mechanical plungers to change their internal cavity volume, resulting in a shift in acoustic properties. To illustrate, Figure 10 displays a metamaterial comprised of pneumatically-actuated Helmholtz resonators, with each unit-cell acting in one direction and arranged periodically across a 2D plane [92]. Both the plunger and resonator walls were manufactured using fused deposition modelling (FDM), with the device scale in the order of centimetres. The pistons are moved by inflatable balloons, with some variation in cavity depth between each periodic resonator cell (cavity depth across the device was regulated within an error margin of 3 mm).

**Figure 11:**
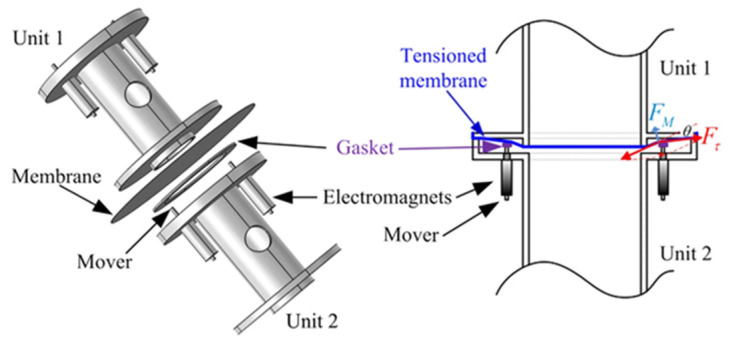
Structure of the active meta-unit with an electromagnetic tuning mechanism. A membrane is tensioned using electromagnetically-operated clamps attached to the pipe exterior [33].

The spectral effects of temperature have been analysed and applied to create a tuneable array of water-filled Helmholtz resonators [29]. By adjusting the water temperature between 0 and 75 °C, the negative bulk modulus band and the local resonant band gap can both shifted by a maximum of 11% from their initial values. This highlights the future possibilities of tuning AMMs via temperature. A promising actuation method uses electromagnets to change acoustic parameters, with an experimental design capable of a 40% resonant band gap shift [33]. The meta-unit design, as shown in Figure 11, consists of an acoustic cavity with a membrane attached to electromagnetic clamps. These clamps, operated with an applied electromagnetic field, manipulate the tension and stiffness of the membrane, consequently changing the acoustic properties of the entire meta-unit.

**Figure 12:**
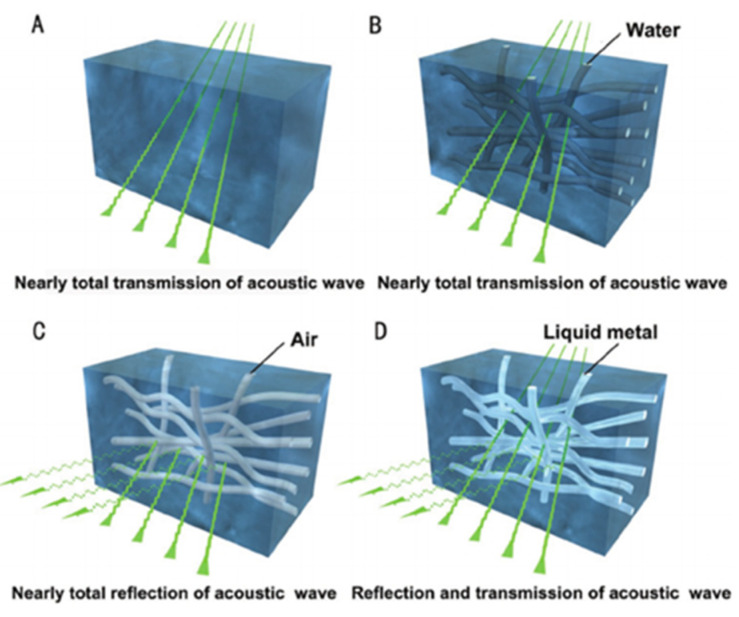
Metagel design consisting of microstructure channels in a tough hydrogel matrix. The acoustic impedance of the hydrogel matches well with water, resulting in almost total transmission of incident waves. These channels can be filled with various liquids to modulate the presenting transmission characteristics of the metagel. In the figure, (**A**) shows “no channels”, with almost total acoustic transmission; (**B**) shows “water-filled channels”, with almost total acoustic transmission; (**C**) shows “air-filled channels”, with almost total acoustic reflection; and (**D**) shows “liquid gallium-filled channels”, with combined transmission and reflection characteristics. (Reprinted from [93], with the permission of John Wiley & Sons Inc.).

Hydrogels are prevalent materials in the literature surrounding active AMMs, broadening the field by introducing soft, flexible capabilities that allow for easier physical actuation. These malleable, hydrogel-based AMMs are referred to as metagels. A novel hydrogel-composite design, shown in Figure 12, includes fillable channels within the hydrogel that can tailor the metagel’s acoustic properties in order to create broadband tuneable transmission/reflection [93]. The internal metagel microstructure can be filled with any fluid material to change the acoustic impedance, resulting in a variety of effects on incident sound waves, as shown in the sub-figures of Figure 12. Mismatched acoustic impedance is a significant challenge to overcome in medical and underwater imaging, and therefore the ability to adapt these properties is a valuable development for the potential applications of metamaterials.

**Figure 13:**
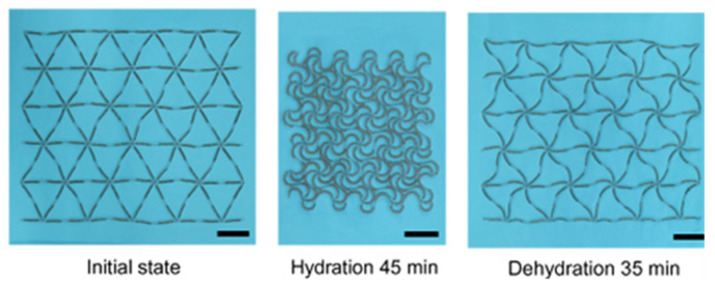
Lattice comprised of hydrogel-composite meta-units, with successive stages of hydration displayed. Here, the scale bar length equates to 5 mm. The initial state is shown at ambient conditions, before immersion in water for 45 min, followed by dehydration via a drying oven set at 75 °C for 35 min [36].

By using hydrogels as a primary design material, this also allows for movement control through changes in hydration. A novel hydrogel-composite metamaterial uses a horseshoe-shaped unit design to facilitate the unusual behaviour of shrinking with water absorption, and vice versa [36]. The microstructure, shown in Figure 13 at various stages of hydration, works by converting hydraulic swelling into bending deformation, resulting in anisotropic negative swelling on the macro-scale. Though hydration, this metamaterial has fully tuneable stress–strain curves and can present specific anisotropic expansion/contraction behaviours based on its geometry. A multi-material polyjet printer was used for fabrication with a minimum layer thickness of 90 μm. In addition, hydrogels are low-cost, environmentally friendly and compatible with human tissue. Manufacturing hydrogel-composite structures with finer detail would create more versatility in the achievable material properties and improve tunability across a wider broadband.

A relevant lattice-type structure here is tensegrity metamaterials, which, through their geometrically non-linear response, can tune global elastic characteristics and hence modulate the structures effect on acoustic waves. These structures consist of an assembly of rigid, compressive elements and deformable, tensile elements [94] that result in complex, non-linear mechanical behaviour determined by the geometric, mechanical and pre-stress variables [95]. Tensegrity metamaterials typically consist of repeated units of tensegrity prisms within an array or lattice. Small changes in the pre-stress of components making up the prism unit-cell result in drastically different acoustic and mechanical properties, which is useful for application in engineering, architecture and acoustic industries. Novel application examples include acoustic lenses [96], impact protection devices [94], mechanical actuators and sensors [97], temporary shelters and deployable antennas [98], most of which require units to be scaled down to be adequate size for feasible use. For example, tensegrity prisms have been utilized to design a tuneable impact-absorption device that, should the prism unit-cells be scaled down to 10 μm, could maintain an effective impact protection barrier with a feasible total length requirement of 10 mm [95].

Active AMMs are designed to have modifiable effects on the surrounding acoustic field; therefore, the changeable parameters are usually directly related to the equations for acoustic wave propagation. Bulk modulus and effective density are of specific interest, with a significant portion of active AMM research focused on allowing these parameters to be tuneable [27,28,30,32,88,99,100]. Many new tuneable devices build upon early AMMs, such as Helmholtz resonator arrays and periodic lattice structures, by incorporating active elements. A meta-unit design was proposed using an actively-controlled system of symmetric, double Helmholtz resonators [101], which successfully tuned the bulk modulus (including negative values) over a broad frequency range by using piezoelectric diaphragms with active feedback control. A theoretical acoustic cloak with tuneable parameters is then discussed with multiple layers composed of these periodic meta-units. The acoustic parameters of each layer can be changed by tuning the gain of the diaphragms, resulting in a fully-modifiable cloak over the frequency range of 3000–6000 Hz. The upper frequency limit is restricted by the subwavelength size the Helmholtz resonators, and so further developing microscale manufacturing methods could help realize the potential of a broadband, fully-tuneable acoustic cloak.

## 3. Additive Manufacturing Methods

The previous section demonstrates the wide range of AMM designs in the literature but is by no means exhaustive. However, it is clear that the concepts and theoretical approaches are being pushed towards greater complexity, and it is our opinion that the greatest barrier to increased complexity will be the ease of manufacture. In this section, we will discuss additive manufacturing methods currently used, which, within the scope of this review, include vat polymerization techniques, powder bed fusion techniques, extrusion and deposition methods, and hybrid techniques.

### 3.1. Vat Polymerization Techniques

The distinctive feature of vat polymerization is that targeted photopolymerization is employed to construct custom 3D parts, as opposed to other methods which use heat-based fusion or material extrusion. These additive manufacturing techniques can be broadly defined as the curing of photo-reactive polymers via an electromagnetic radiation source (for example, lasers, visible light and UV light) [11]. The techniques within the vat polymerization category which are discussed comprise stereolithography (SLA), digital light processing (DLP), and multi-photon polymerization (MPP). Due to the fabrication process, material options are primarily restricted to liquid photopolymer resins; however, small particulates of various materials, such as ceramics [102], metals [103] and live cells [104], can be added to influence material properties.

#### 3.1.1. Stereolithography (SLA)

**Figure 14:**
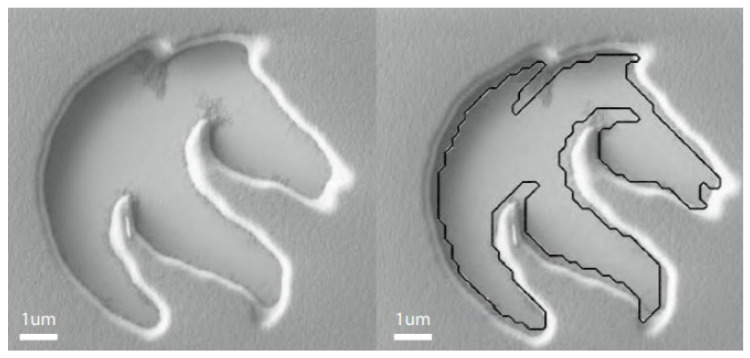
Electron microscope image of a seahorse feature printed using an optimized SLA technique. The right-side image has the predicted shape superimposed. The scale bar here equates to 1 μm [107]. (Reprinted from IFAC-PapersOnLine, Volume 50, Issue 1, A. Fleming, et al., ‘Experimental Scanning Laser Lithography with Exposure Optimization’, Pages 8662-8667, Copyright (2017), with permission from IFAC).

This 3D printing technique was among the first developed and involves the scanning of a photo-curable liquid, layer by layer, to create a 3D part [2]. Traditional SLA cures the 2D cross-section using a laser beam moving in the X–Y plane across the vat of liquid material, before submerging the part one layer-depth and creating the next layer. The laser can use UV or visible light; in some cases infrared light has been used to cure the resin [105]. The exposure time of the laser directly relates to the layer thickness, calculated using the Beer–Lambert law, and microscale cure depths as low as 25 μm can easily achieved by shortening this time [106], where the citation provides a detailed explanation of the SLA printing process. Depending on the material and geometry, lengthy post-processing can be required to maintain mechanical properties. For example, a thermal post-cure increased the compressive modulus of thin polymer layers from 0.6 MPa to 25 MPa, including a 24-h ethanol soak of both samples, which further increased the modulus. The absolute minimum resolution for SLA generally does not exceed 10 μm; however, recent experimental methods employed an optimization algorithm to control the laser beam exposure and consequently achieved resolutions lower than a single micron [107], as illustrated in Figure 14.

In another example of SLA, a small-scale Helmholtz resonator (total length 5.1 mm with a wall thickness 0.5 mm) was successfully fabricated and validated experimentally with a commercially available SLA device [108], the minimum resolution length of which was 27 μm [109]. The resolution limits of SLA methods are enforced from several factors, including the absorption spectra, the focal point size/resolving power of the laser and the level of inhibitor molecules present (usually diffused oxygen) [2].

**Figure 15:**
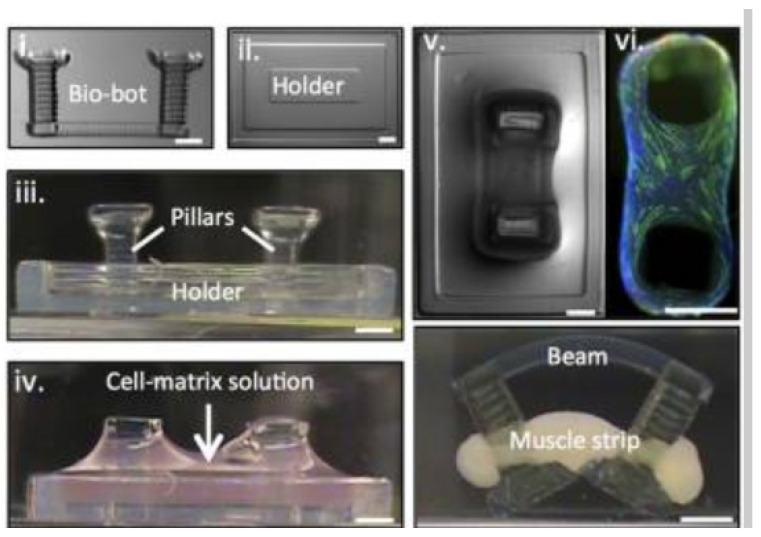
Assembly process of a hydrogel micro-SLA-printed bio-bot, actuated with skeletal muscle. The biobot (**i**) is placed within the holder (**ii**), and the assembly (**iii**) is filled with the cell-matrix solution. This material is compacted around the biobot pillars to form a solid muscle strip (**v**) and with immunostaining (**vi**). The final device is shown in the bottom-right. All scale bars here are 1 mm [104].

Further SLA restrictions exist in the printable material options, which generally are a form of photocurable liquid; however, there is still versatility, with ceramic inclusions being used to improve mechanical, thermal, electrical and magnetic properties of the base resin [102]. Additionally, to print pure, non-composite ceramic material, there must be a process to debind and remove the photocurable resin. Despite these restrictions, there are a variety of materials printable using SLA. A novel example is a millimetre-scale bio-bot comprised of hydrogel and actuated by skeletal muscle [104], as shown in Figure 15.

SLA printers have been utilized to fabricate devices for acoustic and ultrasonic applications. Notably, an in-air ultrasonic beam shifter was produced from clear resin, using a commercially available 3D SLA printer (Asiga PICO2 HD), where the prototype was experimentally verified to confirm its beam shift performance on acoustic fields [110]. The acoustic response of the device was tested and compared with a simulated model in COMSOL Multiphysics, where the results of both were largely in agreement with the analytically-predicted behaviour. This metamaterial device was composed of periodically-spaced plates, with a thickness of 500 µm, and was appropriately scaled to operate at 40 kHz.

**Figure 16:**
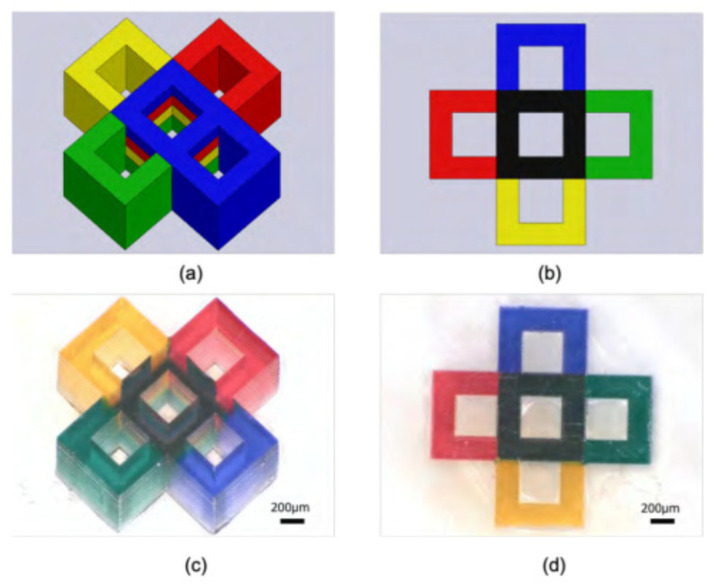
Micro-SLA-fabricated 3D cross-shape, printed with multicolour resins. The modelled **CAD** geometry is shown from orthogonal (**a**) and top-view (**b**) angles alongside the finished part at orthogonal (**c**) and top view (**d**) angles. Here, scale bars are 200 μm [111].

Multi-material micro-SLA has also recently been demonstrated, with several coloured resins used in a single print and a layer thickness of 30 μm [111], illustrated in Figure 16 for the multi-colour print at 200 μm scale. An active AMM has also been fabricated with ferroelectric material, allowing tuneable stiffness via an external electric field [112]. The efficiency of this method is limited due to the scanning mechanism, which is required to move across the vat incrementally to cure a layer. Other vat polymerization methods address this drawback, namely digital light processing (DLP), by curing the entire layer in one exposure. Alternatively, better resolutions approaching the nanoscale can be achieved with multi-photon polymerization, another vat-polymerization technique.

#### 3.1.2. Digital Light Processing (DLP)

From SLA technology, another microscale printing method emerged—digital light processing. This is similar to SLA; however, DLP cures an entire layer at once by projecting patterns of light [113]. This method features both high resolution and quick print times, with resolution lengths as small as 0.6 μm and a layer printing speed of 1000 mm/h. Some DLP printers are capable of printing large volume parts in short timeframes, where for example a part with dimensions 38 cm by 61 cm by 76 cm was reportedly printed in 1 h and 45 min with DLP. However, fast printing at such size scales incurs the cost of lower precision and resolution.

**Figure 17:**
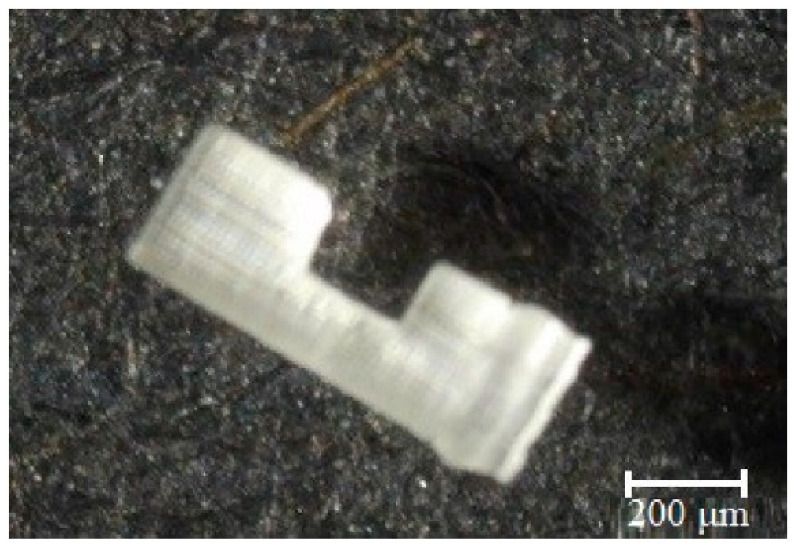
A micro-beam fabricated with DLP using clear IP-S resin photopolymer, where the smallest non-support feature is 86.7 μm. Twenty supports with a diameter of 3 μm were used during the building process. Here, the scale bar is 200 μm [114].

Notably, a micro-beam was fabricated using DLP with a layer thickness of 30 μm and an exposure time of 3 s (per layer) [114]. The printed micro-beam is displayed in Figure 17, taken from a microscope image. To prevent breakage, structural supports were added, with a diameter of 3 μm and a height of 5 μm. The same beam design was also fabricated using SLA, but the latter method was not able to print the supports, so instead the beam was printed in 2D. This direct comparison demonstrates DLP is not only the faster and more precise method, but the micro-resolution allowed supports to be added that vastly improved part quality. DLP thrived at printing complex, microscale 3D structures, while micro-SLA performed better with simpler 2D structures.

**Figure 18:**
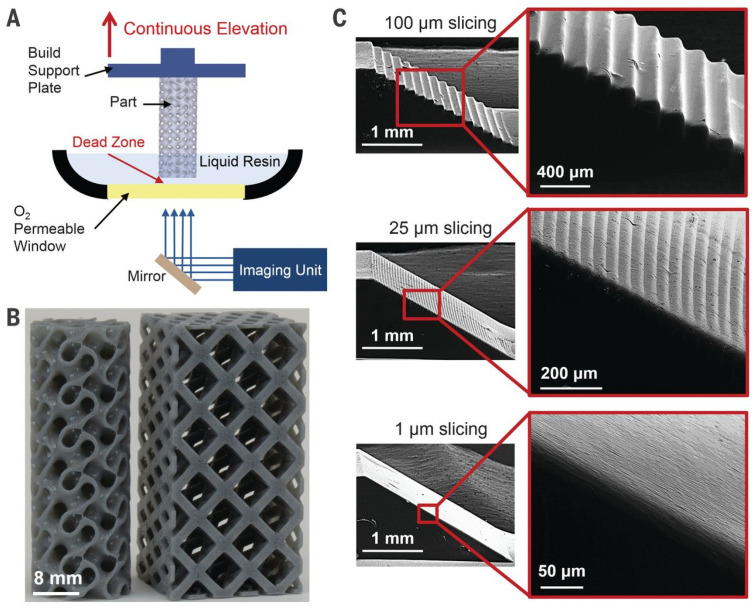
Gyroid structure printed in microscale with DLP showing (**A**) a diagram of the DLP set-up; (**B**) the gyroid part, printed at 500 mm/h; and (**C**) ramp test prints with slice thicknesses of 100 μm, 25 μm and 1 μm, successively. (From [115], Reprinted with permission from AAAS).

In the literature, SLA and DLP are further contrasted, with SLA’s printing speed (in the vertical Z direction) restricted to several mm/h due to its stepwise regime, where each individual layer takes seconds to manufacture and is 50–100 μm thick. Unlike SLA, DLP’s printing is generally continuous and restricted instead by resin cure rates and viscosity, and hence can achieve speeds at least two orders of magnitude faster [115]. A gyroid structure printed at 500 mm/h using DLP can be seen in Figure 18B, with a diagram of the DLP process. An important benefit of this manufacturing technique is that increasing resolution/layer thickness has less effect on the print time—this was tested with the ramp structures shown in Figure 18C printed with slice thicknesses of 100 μm, 25 μm and 1 μm. There are additional factors to consider for viable printing, such as modulating the “dead-zone” and oxygen flux through the permeable window.

**Figure 19:**
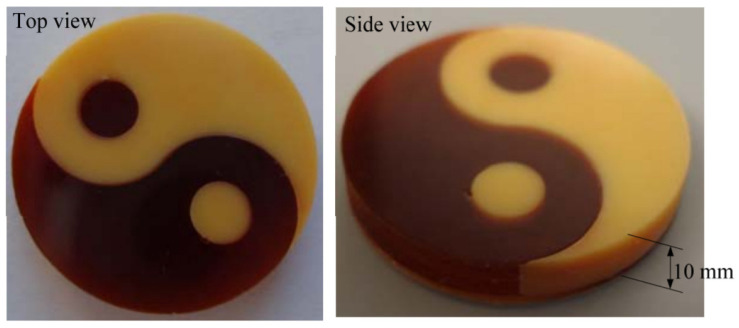
Test print for a multi-material DLP method using red and yellow photocurable resin (Reprinted from [116], with author permission).

The printable material is limited by similar restrictions to SLA; additionally, multi-material prints are more difficult to achieve with DLP because of the holistic nature of the curing mechanism. One of the earliest DLP multi-material prints, in 2011, required the material vat to be removed and replaced to print a different material [116]. This method was particularly susceptible to material waste and material contamination between vats, which was countered by using a bottom-up projection method and implementing a two-stage cleaning process between vat changes. This adapted DLP method managed to print multi-material parts with a layer thickness of 100 μm, as illustrated in Figure 19.

A subsequent multi-material DLP method, with a top-down approach, employed an air jet-based cleaning step to minimize resin waste and cross-contamination [117]. This novel method forewent the use of a vat, instead utilizing a glass plate with different photocurable liquids applied in “puddles” using electronically-controlled syringes. Two materials can be printed simultaneously, with a wide variety of resins able to be utilized. A layer thickness of 25 μm was used, and multilateral layers reportedly took (in ideal conditions) 2.2 times longer to print than a single material equivalent. To shorten the time taken to switch materials in multi-material DLP printing, another (bottom-up) method was proposed that uses dynamic fluid control to exchange resins [118]. Photo-polymerization takes place in a sealed fluidic cell, with UV light exposed through a transparent top window, in which different liquid materials are pumped through and directed using a series of valves. Resins with polymer, ceramic and metallic composites can be used, as well as active hydrogels, allowing thermally-actuated soft robots to be printed. A full material change was reportedly 30 times quicker than manually changing material vats, with four measured materials exchanging 95% cell volume in 3.2–12.8 s. Furthermore, this method demonstrated high resolution, with a minimum feature size of 5 μm.

**Figure 20:**
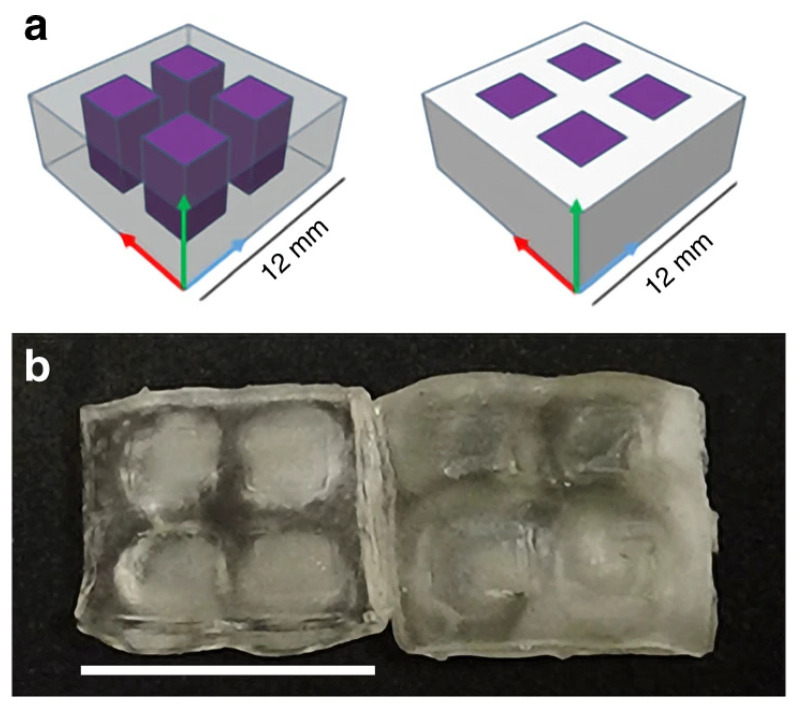
Multi-material printed part using DLP with UV and visible light exposure, where (**a**) shows the geometry of the part with UV irradiated sections highlighted in purple, and (**b**) shows the finished print with (**left**) no thermal post-cure and (**right**) 3-h thermal post-cure at 60 °C. Here, the scale bar represents 12 mm [120].

There are currently promising advancements in printing metallic materials with DLP by using silver nanoparticles incorporated in a polymer matrix [103]. Another novel material is hydrogel, often used in active metamaterial designs. A temperature-responsive hydrogel was successively printed in the microscale using DLP [119]. Some multi-material structures have been experimentally produced by exposing the same base resin to either UV or visible light, hence creating regions with different material properties [120]. The geometry of an example part printed using DLP is shown in Figure 20, where the purple areas are rendered with UV light and clear areas with visible light. Another proposed multi-material DLP approach achieves a minimum feature size of 200–300 μm [121]. Overall, the resolution seems to be significantly reduced when using multi-material capable technologies, with printed designs struggling to reach the microscale.

**Figure 21:**
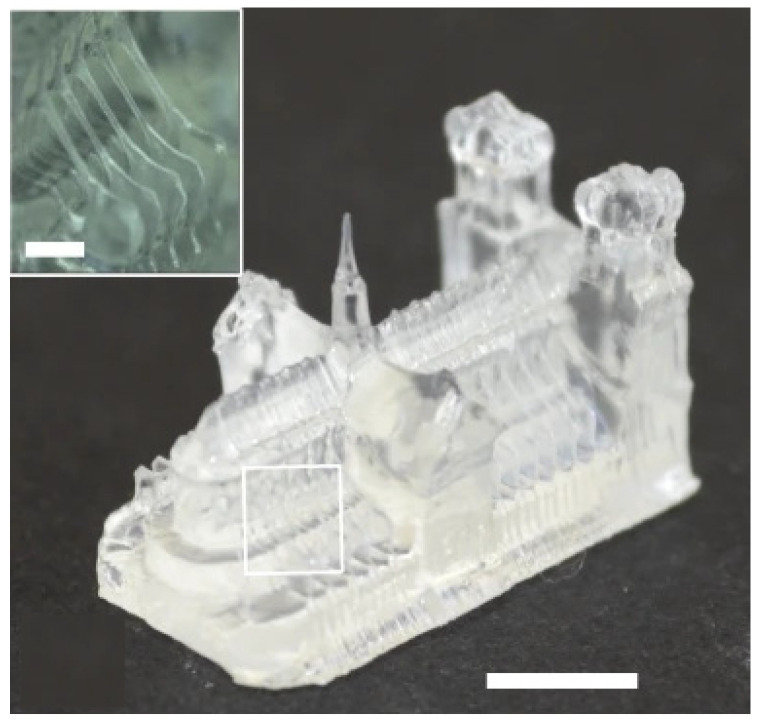
“Notre Dame” printed part using the novel tomographic multi-beam method. The scale bars are 5 mm in the main image, and 1 mm for the inset [122].

An emerging tomographic technique building on DLP technology employs a single-step multi-beam exposure, omitting the typical stepwise protocol of most additive manufacturing methods [122]. This unique polymerization approach requires the resin to be transparent and the subsequent absorption length to be tuned to ensure that light can reach the necessary cure depths. The transparent vat of resin is rotated, and a DLP modulator displays low-energy light patterns in sync with this rotation, and after a full revolution, a 3D rendered part is produced. The intensity of a single illumination pattern is insufficient to cure the resin; hence, the summation of every light pattern is required to solidify the finished part. This new method has a theoretical resolution of 23–33 μm and has successfully printed structures with microscopic features. Figure 21 shows a miniature “Notre Dame archway” printed in acrylic resin, with a smallest feature size of 80 μm and a relatively very short print time of 19.5 s.

#### 3.1.3. Multi-Photon Polymerization (MPP)

First proposed in 1997 [123], multi-photon polymerization (or two-photon polymerization) is an advancement on the original SLA fabrication method, the key distinction being that while SLA cures a layer with incremental laser exposure, MPP cures a layer point by point. The MPP fabrication process involves directing a femtosecond laser pulse through a lens, so it converges into a single focal point within the vat. This focused energy triggers the two-photon absorption process, and a small volume of resin is polymerized as a result. The volume pixel (voxel) of cured material can be several orders below the laser wavelength, which is typically about 810 nm [2]. This method allows for an extremely precise curing of resin and hence offers a very high resolution, with a minimum resolution size in the order of 100 nm [113]. This does come at the cost of print size, with X–Y maximum print sizes ranging from 10 μm by 10 μm, to 2.2 mm by 2.2 mm. Similarly to DLP, MPP is not limited by supporting materials or layer-by-layer fabrication regimes, as polymerization can occur in any 3D spatial point, and not just the current build surface [7]. These characteristics make MPP of significant interest as a fabrication method for drug-delivery devices, implantable microelectromechanical systems (MEMS) and tissue scaffolding applications.

**Figure 22:**
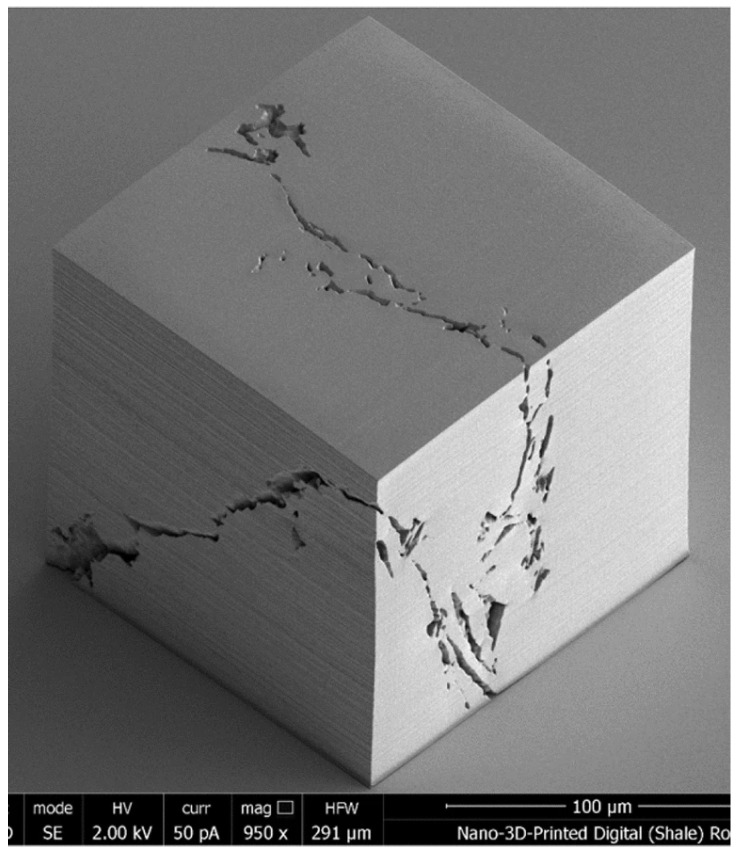
MPP-printed 150 μm^3^ cube of replica shale rock with nanoscale features, where the image is taken with an electron microscope [15].

MPP in prior studies has repeatedly demonstrated nanoscale print size, succeeding the aforementioned vat polymerization methods significantly in ultra-high resolution [124,125]. An example of this is a synthetic nanoporous geomaterial fabricated with MPP, as illustrated in Figure 22, which shows an electron microscope image of a 150 μm^3^ cube of replica shale rock with a minimum feature size of 160 nm [15]. The print was evaluated with the microscope and has no external defects, validating the quality of this 3D printing method. Feature widths as small as 65 (±5) nm were consistently achieved by lowering the laser wavelength to 520 nm.

**Figure 23:**
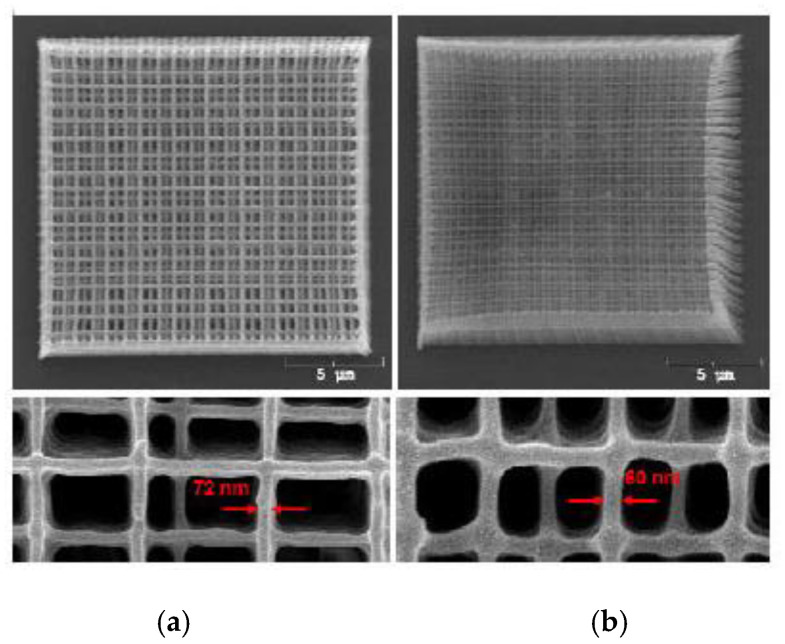
Nanoscale woodpile structures printed with MPP showing (**a**) laser excitation of 0.6 μW and a resultant beam thickness of 72 nm, and (**b**) laser excitation of 0.45 μW and a resultant beam thickness of 60 nm [126].

In another example of MPP fabrication, Figure 23 displays two woodpile structures with beam thicknesses of 72 nm and 60 nm, respectively [126]. Due to the high spatial resolution of MPP, there are limitations with regards to print speed. MMP printing speeds generally range between 0.5 mm/s to >100 mm/s in the X–Y plane [127,128,129,130,131], depending on the resolution required and technology used. Novel microscale methods incorporating high frequency resonant-imaging mirrors can reach speeds as high as 8000 mm/s [132]. It is important to note that these speeds are in the X–Y plane and, as this method cures each voxel individually, its speed in the Z-direction is severely limited, making this method only suitable for small or 2D prints.

The manufacture of small-scale lattice structures is a significant area of interest for tensegrity metamaterial research. Discussed earlier in Section 2.2.3, tensegrity structures employ compressive and tensile elements to produce devices with tuneable and non-linear mechanical and acoustic behaviours. These structures require very small feature sizes to maintain complex dynamic behaviours and to be deployed in various applications. MPP offers a solution to these manufacturing limitations and has been used to build bi-stable tensegrity lattices with nanoscale features [97]. The process used a diffusion-assisted femtosecond laser for high-precision polymerisation and enabled the effective fabrication of unit-cells and arrays comprised of struts with a 250 nm radius. Microscopic imaging was used to assess the deformation phenomena at nanoscales and their effect on macroscopic force–displacement curves. The analysed nanolattices successfully demonstrated a bi-stable response, viscoelastic behaviour and softening and stiffening deformation mechanisms. Therefore, MPP was shown to be a viable fabrication method for tensegrity metamaterials.

**Figure 24:**
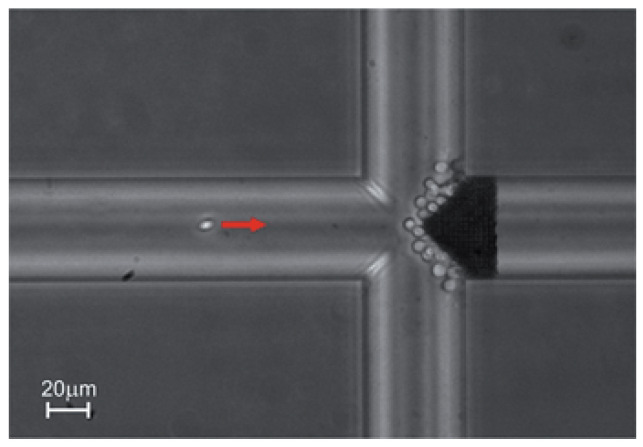
Microscope image of red blood cells filtered from plasma with a micro-porous filter, printed with MPP [138]. (Republished with permission of Royal Society of Chemistry, from [Lab on a chip, L. Amato, et al., Volume 12, Issue 6, Pages 1135-1142, 2012]; permission conveyed through Copyright Clearance Center, Inc).

While the printable resin needs to be photocurable, nanocomposite inclusions can be added that allow for a variety of material properties, such as conductivity, magnetism, piezoelectricity, mechanical properties, high refractive index [128] and biodegradability [133], which is also true for other vat polymerization methods. Soft materials, like hydrogel, can also be printed for use in active metamaterial micro-devices [134]. There is significant interest in MPP printing of hydrogels for drug delivery and tissue scaffolding, due to the method’s high spatial resolution and composite-printing capabilities [135,136]. Other medical applications for this printing method have been realized [137], for example with an MPP-printed, porous filter being used to filter blood cells in a microfluidic chip [138]. A microscope image of red blood cells filtered from plasma with a filter printed using MPP is shown in Figure 24.

#### 3.1.4. Summary/Manufacture of AMMs

Vat polymerization methods are prolific for constructing AMM prototypes, with several examples presented in the previous AMM section in Figure 6 and Figure 8a,b. In this section, a variety of novel fabrication examples were discussed, demonstrating the capabilities and limitations of three vat polymerization methods: SLA, DLP and MPP. Below, the variables pertaining to the additive manufacture of AMMs for use in widespread industry are discussed, with the aforementioned techniques compared and contrasted for suitability in each category. In general vat polymerization is rapid and accurate with cm-scale build volumes. The use of a liquid to cure to solid means that certain features, such as inclusions, can be difficult to print, as liquid can remain trapped if a means of exit is not included, which increases post-processing.

**Ability to mass-produce:**

In terms of vat polymerisation techniques, DLP is the fastest production method by a significant margin. Additionally, its simultaneous layer curing regime also allows for the creation of several printed parts without additional printing time. SLA is the next fastest, due to its layerwise scanning, with MPP being the slowest fabrication process because of its precise point-by-point curing mechanism. Unlike DLP, printing extra parts adds proportionally to the printing time for SLA and MPP. Considering this, DLP is the most promising vat polymerisation method for mass-producing AMMs.

**Minimum printable feature size:**

MPP has the highest resolution of all the additive manufacturing techniques discussed in this review, with a minimum feature size well within the nanoscale (as low as 60 nm). It also is not restricted by the use of support structures, as the point-by-point polymerization regime can fabricate at any 3D spatial point, unlike DLP and SLP, which are limited to a 2D build surface. DLP performs better than SLA at printing small features, but with the resolution of both generally restricted to the microscale. DLP has been recorded to reach minimum features sizes as small as 600 nm. With all systems, the build volumes are typically inversely correlated with build volume, meaning higher resolution comes at the cost of smaller finished products.

**Multi-material printing:**

Multi-material printing is inherently difficult with vat-polymerisation techniques due to the possibility of cross-contamination of the different liquid resins used as build material. Despite this, several multi-material prints were presented in this section, with SLA presenting the best multi-material capabilities in Figure 16. DLP also presented multi-material methods requiring either intensive cleaning steps between vat changes to ensure no cross-contamination of resin, or complex and novel implemented systems for resin transfer (see references [117,118]). Overall, SLA provides the simplest and most effective multi-material printing process out of the vat polymerisation techniques; however, extrusion/deposition techniques perform significantly better in this category.

**Active metamaterial fabrication:**

The construction of active metamaterials is closely linked to the range of build materials available to the applicable fabrication methods. Printed active metamaterial examples using vat polymerisation methods are sparse in the literature, with no AMM-specific examples mentioned in this text. The material restrictions of the photocurable liquid resin hinder the fabrication of actuatable metamaterials, making it generally unfeasible to produce these with any vat polymerization techniques. The exception to this is hydrogel-based active designs, as these can be quite easily fabricated using SLA, DLP and MMP, with an SLA-printed example shown in Figure 15.

### 3.2. Powder Bed Fusion Techniques

Powder bed fusion is an additive manufacturing method wherein a heat source is applied to a bed of fine particles in order to bind that construction material together and form highly-detailed 3D parts [139]. The scope of this review is to consider fusion methods using lasers (SLS/SLM), electron beams (EBM) and infrared projections (MJF). Interest in this type of additive manufacturing has grown in recent years due to its low cost, material variety, and its requirement for no or minimal printing supports. Due to the powder form of the construction material and heat based bonding method, a wide range of material types can be used, including plastics, glass, metals and alloys, with the unused powder also being recyclable.

#### 3.2.1. Selected Laser Sintering/Melting (SLS/SLM)

These two powder bed fusion methods use laser beams to bond powder into 3D parts. However, there is a key distinction between SLS and SLM. The former works solely with plastics, whereas the latter primarily with metals [139]. The manufacturing process is of a step-wise configuration, with a layer coating of powder sprayed on after every previous bonded layer. For SLS, the fabrication process begins similarly to other printing techniques, where the 3D CAD part is divided into 2D layers with incremental thicknesses, and the printing chamber is prepared with the desired powder base. Next, a CO_2_ laser heats the layer, point by point, until the desired pattern is sintered together. The platform descends in the Z direction, and a new layer of powder is spread over evenly and the process repeats until the part is finished. SLM operates a similar way but with some important differences. In order to melt the material, the laser must be higher energy, also requiring inert gas to function. Overall, SLM has several benefits over SLS, namely it is faster and has a lower minimum layer thickness (20 μm for SLM compared to 80 μm for SLS). SLS can print with nylon, thermoplastic and polystyrene, while SLM can print with metallic powders. Recent developments that have incorporated optical fibres with high-powered lasers have enabled further material options for SLM, with composite and ceramic printing now within reach. Both SLS and SLM can yield high quality prints; however, post-processing is required to eliminate potential issues such as porosity, surface roughness and crack defects. SLM can incur welding defects, so it requires thermal post processes to assure part quality.

**Figure 25:**
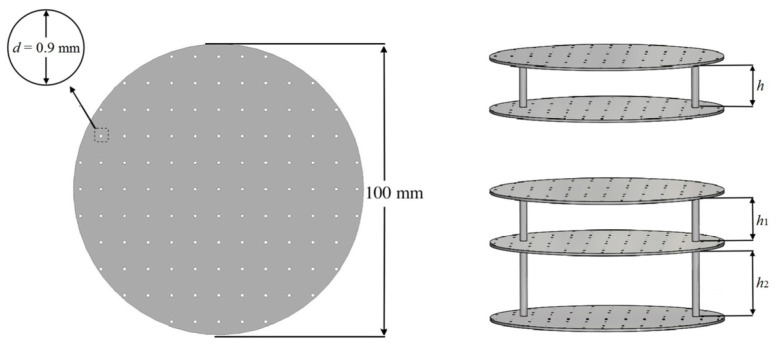
Geometry of a micro-perforated plate fabricated with SLS [140].

SLS can print with relatively high spatial resolution and short print duration; however, SLS generally requires one of these qualities to be prioritized over the other. An example of this is perforated plates for tuneable AMMs [140], as shown in Figure 25, fabricated with a relatively fast printing speed (4000 mm/s in the X–Y plane) and a comparatively large minimum resolution length (0.12 mm layer thickness).

**Figure 26:**
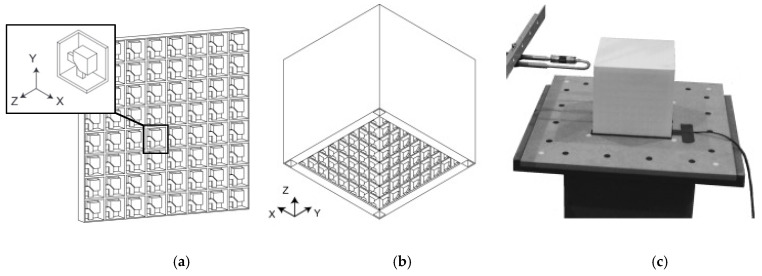
A lightweight panel-based AMM structure, with each panel comprised of 8 by 8 locally-resonant unit-cells and fabricated using SLS, showing (**a**) the schematic of a single panel, with an inset section displaying the geometry of a single unit-cell, (**b**) a diagram of the prototype, where the panels are arranged in a box shape and then housed by a solid material border, and (**c**) the experimental setup to measure the acoustic response of the printed prototype [141]. (Reprinted from Mechanical Systems and Signal Processing, Volume 70, C. Claeys, et al., ‘A lightweight vibro-acoustic metamaterial demonstrator: Numerical and experimental investigation’, Pages 853-880, Copyright (2016), with permission from Elsevier).

An example of an SLS-fabricated AMM in the literature is a locally-resonant panel structure that demonstrates acoustic band gaps [141]. This structure exploits an isolated bending mode that can be easily modelled with an MSD system for straightforward calculation of the band gap frequency. A design was fabricated and tested to demonstrate proof of concept, with SLS specifically chosen as the manufacturing method due to its ability to construct complex topology without the need of support structures. The main limitation of this and other powder bed fusion methods is that the design must not have sealed cavities, otherwise the unused powder cannot be removed. Shown in Figure 26, the AMM prototype contains 8 by 8 unit-cells with a maximum component thickness of 4 mm (minimum possible thickness of 80 μm). The prototype is lightweight, with only 30% weight of each unit-cell being the resonant structure and the rest being the solid enclosure, and modular, meaning the cell configuration of the panels can be changed to produce different acoustic responses.

**Figure 27:**
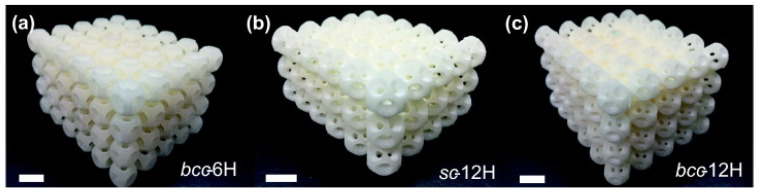
Three distinct printed designs of a 3D soft, auxetic lattice structure, commonly referred to as a Bucklicrystal. These crystal lattices all were SLS-printed using TPU powder and with 60% porosity and show (**a**) a crystal lattice with body-centred-cubic unit-cells with 6 holes, (**b**) a crystal lattice with cubic solid-centred unit-cells with 12 holes, and (**c**) a crystal lattice with body-centred-cubic unit-cells with 12 holes. Here, the scale bar equates to 1 cm [142]. (Reprinted from Materials & Design, Volume 120, S. Yuan, et al., ‘3D soft auxetic lattice structures fabricated by selective laser sintering: TPU powder evaluation and process optimization’, Pages 317-327, Copyright (2017), with permission from Elsevier).

SLS can also be used to print soft AMMs with mechanical actuation capabilities. Several configurations of a 3D soft auxetic metamaterial were fabricated using SLS, as shown in Figure 27, and their resulting mechanical characteristics were investigated [142]. The printed lattice was constructed from thermoplastic polyurethane (TPU) powder, with the material evaluated based on its flowability, size distribution, thermal transition identification and melting and decomposition energy analysis. The final TPU powder was characterized in order to optimize the printing of complex 3D geometries for a specific laser calibration. These lattice structures, known as Bucklicrystals, are designed to exhibit buckling behaviour under compressive loading. This results in volume shrinkage across two transverse directions, and hence, the Bucklicrystals possess a negative Poison’s ratio. Such structures could be used in applications requiring mechanical damping, energy absorption and mechanical actuation.

**Figure 28:**
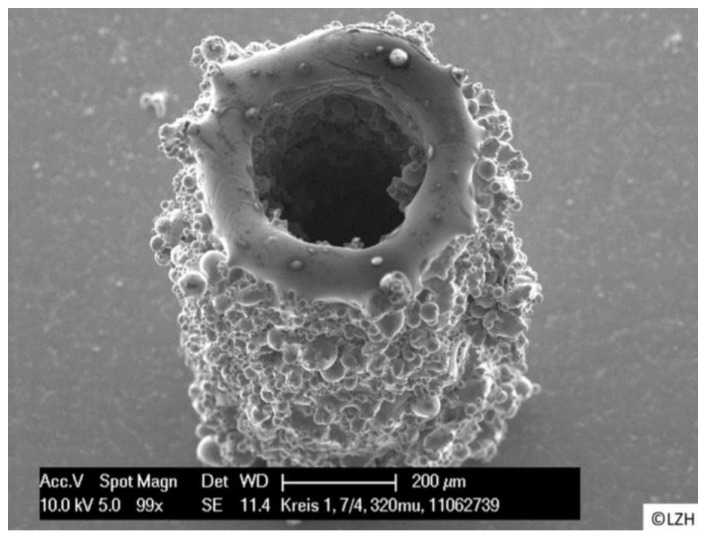
An SLM-fabricated micro-needle for drug delivery applications, where the needle was constructed from 316L stainless steel powder, with a length of 550 μm and an interior aperture measuring 160 μm across [145].

SLM is one of the most promising 3D printing methods for fabricating metallic small-scale parts without the use of composite materials, resins or binders [143]. Key parameters currently restricting SLM to primarily macro-scale fabrication are laser calibration (particularly laser type, intensity and spot size), scanning regime (including strategy, hatch spacing and scanning speed) and powder bed characteristics (such as recoating mechanism and powder properties). Commercial SLM systems use powder particle sizes in the 20–50 μm range, which results in minimum layer thicknesses ranging between 20 μm and 100 μm. Typically for microscale SLM printing, the powder gain sizes must be less than 10 μm, and the laser focal diameter lower than 40 μm [144]. One of the early potential applications of microscale SLM was manufacturing microneedles for drug delivery. A stainless steel needle produced with an inner hollow diameter 160 μm, achieved using a femtosecond laser, is shown in Figure 28 as an example [145]. As observed in the figure, the largest detriment of this print is the outer surface roughness and residue powder, which was not improved after 35 min of ultrasonic cleaning.

**Figure 29:**
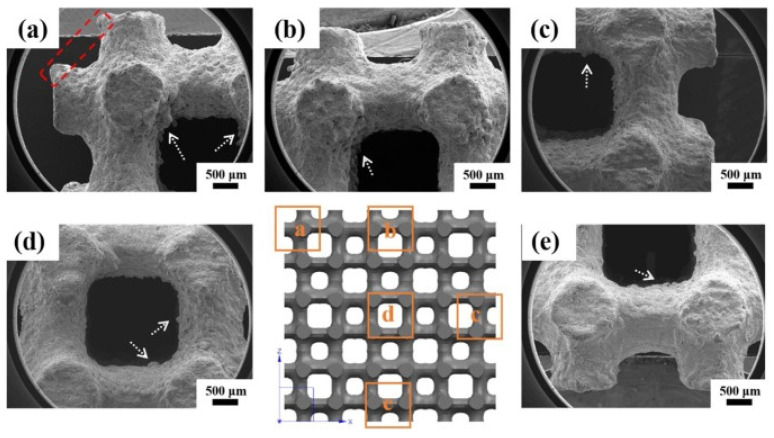
Cross-section of optimized lattice design printed using SLM with Ti-6Al-4V powder. The bottom middle panel displays the pattern geometry with two alternating periodic spacings. The remaining panels (**a**–**e**) respectively show labelled and magnified images of the lattice in the microscale, where the scale bar is 500 μm [148]. (Reprinted from Journal of Manufacturing Processes, Volume 56, L. Zhang, et al., ‘Topology-optimized lattice structures with simultaneously high stiffness and light weight fabricated by selective laser melting: Design, manufacturing and characterization’, Pages 1166–1177, Copyright (2020), with permission from Elsevier).

Recent studies have been conducted on the design and testing of lattices fabricated by SLM [146,147]. In particular, a cubic lattice design was topologically optimized to show increased mechanical properties and energy absorption capabilities [148]. Along with enhanced topology, the implemented improvements include optimizing the printing parameters to limit the prevalence of metallurgical defects, which affect mechanical properties and part quality. The following parameters were used to fabricate a titanium-alloy lattice with periodic spacings of 1 mm and 1.74 mm: (i) a laser power of 280 W; (ii) a scanning speed of 1200 mm/s; (iii) a layer thickness of 30 μm; (iv) a hatch distance of 140 μm; and (v) a scanning direction rotated 67° between alternate layers. This optimized lattice is shown in Figure 29, with no macro defects occurring during fabrication and good surface quality. The final printed result proved to be a high-performance and lightweight matrix structure, fabricated with an estimated dimensional accuracy of +55 μm/−49 μm.

A novel application of printed AMMs is ultrasound cloaking technology, designed to mask the presence of objects from the surrounding acoustic field. SLM has been used to construct an AMM underwater cloak with an operational bandwidth of 100–900 kHz, frequencies commonly used in medical ultrasound and non-destructive testing (NDT) [149]. This broadband ultrasonic cloak uses metagrating—defined as a rigid surface with periodically modulated grooves—to enable its wave-redirection mechanism. The design is simplified so that performance is dependent on a single geometric variable, therefore eliminating the complexity found in conventional underwater cloak designs. SLM was used to construct the cloak out of stainless steel with a periodic spacing of 3.5 mm and a minimum feature size of 1.2 mm. The metagrating proved extremely capable of concealing objects of arbitrary shape and considerable size from incident ultrasonic wavefronts. While this is significant progress in the field of underwater cloaking, further research needed to realise omni-directional characteristics. Further reducing the minimum printable feature size for the SLM process would allow for future innovation in underwater cloaks such as this. For example, reducing the periodic spacing would allow for ultra-broadband capabilities at even higher frequencies.

#### 3.2.2. Multi Jet Fusion (MJF)

This innovative technique builds on the traditional SLS method and shows promising improvements in some key printing variables [150]. While the initial powder bed set up and core construction materials are similar to SLS, the method diverges after this point. Multi jet fusion (MJF) uses an array of jets to dispense a fusing agent on top of the powder bed in the shape of the layer cross section. Following this, instead of lasers, MJF uses projections of infrared light to simultaneously heat and bind an entire layer. This method has many benefits over SLS, with MJF-printed parts demonstrating faster print times and more dimensional accuracy while using effectively-identical polyamide powder. However, the SLS-printed parts exhibit superior mechanical properties, exceeding the MJF-printed parts in Young’s modulus, impact toughness and tensile strength. This is likely due to SLS being more developed. SLS techniques have been improved over decades and the optimal laser parameters for commonly-used materials are well understood. MJF is an emerging technique and will take more time in development before its printing abilities are optimized, in which case it shows potential to surpass SLS and several other printing methods in the future.

**Figure 30:**
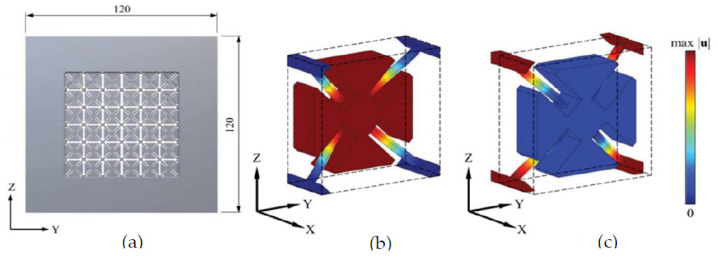
The design of an acoustic metamaterial panel that uses local resonance to attenuate sound, where the panel consists of a 2D array of unit-cells held in place by a solid border. These unit-cells consist of a clover-shaped mass inclusion connected to thin structural supports. The figure shows (**a**) the schematics of the panel design, with dimensions shown in mm, (**b**) the displacement plot of a single meta-cell during attenuation peak at 1050 Hz, and (**c**) the displacement plot of a single meta-cell during transmission peak at 1380 Hz [151]. (Used with permission of American Society of Mechanical Engineers, from [151] [‘Experimental and Numerical Assessment of Local Resonance Phenomena in 3D-Printed Acoustic Metamaterials’, Journal of vibration and acoustics, D. Roca, et al., Volume 142, Issue 2, 2020.]; permission conveyed through Copyright Clearance Center, Inc.).

MJF was used to print an acoustic metamaterial panel capable of attenuating sound at a specific frequency range [151]. The MJF-printed metamaterial panels, as shown in Figure 30a, were compared experimentally to solid panels of the same material (polyamide 11) with similar dimensions. The AMM panels demonstrated superior sound attenuation over their homogeneous counterparts. Aside from the attenuation peak at 1050 Hz, the metamaterial showed sound transmission peaks at 550 Hz and 1250 Hz, which mark the limits of the band gap region. Finite element analysis (FEA) models of the attenuation and transmission peaks are shown in Figure 30b,c, respectively. The resonant modes were skewed by up to 200 Hz due to the geometric tolerances of the fabrication method, with 0.1 mm deviations in the meta-cell diameter affecting the frequency response.

The internal resonance of the system is very sensitive to small deviations in geometry, which is a common problem in the fabrication of resonant AMMs. Therefore, refining the accuracy of powder bed fusion printing methods could be a significant step towards the large-scale production of feasible locally-resonant metamaterials for industrial application.

#### 3.2.3. Electron Beam Melting (EBM)

This fusion method uses an electron beam as the heat source in order to fabricate both metal and plastic material formulas [139]. The powder bed is prepared similarly to the previous methods, and an electron beam is deployed from a tungsten filament and controlled via a computerized coil. While there is more variety in the possible fabrication materials, this method does require a vacuum-sealed build chamber to function.

**Figure 31:**
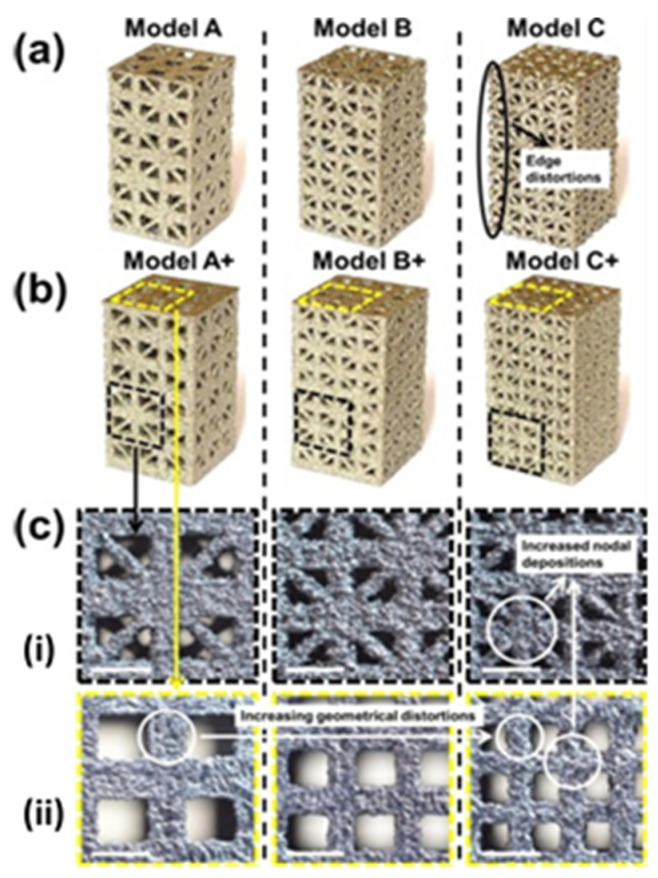
Digital images of three different micro-lattice designs (**a**) with and (**b**) without solid outer supports on the edges. The unit-cells of Models A to C are progressively smaller in size, with cell lengths ranging from 3.33 mm to 2 mm. The supports for models A+ to C+ also range in thicknesses from 670 μm to 400 μm. Sub-figure (**c**) shows magnified sections of the models displayed in (**a**,**b**), with focus on the smaller (**c**)—(**i**) and larger (**c**)—(**ii**) pore structures. Here, the scale bar is 3 mm [152]. (Reprinted from Additive Manufacturing, Volume 36, P. Wang, et al., ‘Electron beam melted heterogeneously porous microlattices for metallic bone applications: Design and investigations of boundary and edge effects’, Copyright (2020), with permission from Elsevier).

A recent example shows EBM used to fabricate a metallic micro-lattice with mechanical capabilities that can replicate bone [152]. Both EBM and SLM are of interest in biomedical applications due to their ability to fabricate biocompatible metallic structures with high complexity; however, EBM has more material options than other powder bed methods. The lattice design incorporates large and small size pores heterogeneously throughout the structure for the purposes of nutrient flow and cell seeding, respectively. A compressive strength of 169.5–250.9 MPa was achieved by the printed structures, along with a Young’s modulus of 14.7–25.3 GPa and high porosity (up to 60%). This successfully imitates the strength of bone, which has a compressive strength in the region of 188–222 MPa and a Young’s modulus in the order of 15–35 GPa. The addition of solid edges to the micro-lattice resulted in an increase of 45% in compressive strength and 25% in Young’s modulus. Several printed samples are shown in Figure 31 with different dimensions, with the pore architecture highlighted in Figure 31c. A matrix wall thickness of 500 μm was used with a high relative density of 50% to ensure no buckling under low strains. Ti-6Al-4V powder was used to construct the lattices, with an average particle size around 70 μm, and the following parameters were implemented in the EBM setup: (i) 60 kV accelerating voltage; (ii) 50 μm layer thickness; (iii) a line offset of 100 μm; (iv) a hatch depth of 50 μm; and (v) the vacuum chamber maintained at 2 μBar. Some challenges with geometry distortion occurred when feature sizes reached 400 μm, as this is near the resolving power of EBM.

**Figure 32:**
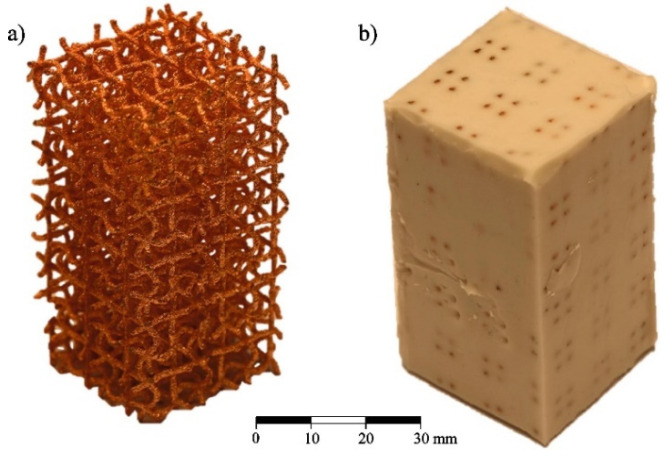
A uniform, chiral structure made of copper shown (**a**) without and (**b**) with a rubber encasing. The hybrid composite structure (**b**) combines the initial EBM-bonded copper lattice with a silicon polymer filler while maintaining a vacuum seal of 0.5 MPa in the build chamber during fabrication [153]. (Reprinted from Composite Structures, Volume 234, N. Novak, et al., ‘Mechanical properties of hybrid metamaterial with auxetic chiral cellular structure and silicon filler’, Copyright (2020), with permission from Elsevier).

Given the powder bed can only contain one fusible material at a time, multi-material parts are usually comprised of one base powder (typically metallic) and another non-fusible support powder/material. A novel multi-material design was proposed consisting of an auxetic chiral meta-cell that is permeated with silicon to make a hybrid material [153]. The chiral shape used corresponds to the tenth eigenmode of the regular cubic unit-cell, the behaviour of which is well known in the literature. Uniform structures of the unit-cell are fabricated via EBM using copper powder, with some prototypes kept in this condition (without the silicon composite) as a point of comparison for the performance of the hybrid structures. An example of this is shown in Figure 32. The hybrid design was found to reach much higher values of plateau stress during compressive testing to failure. In summary, the introduction of the silicon support material enhanced the mechanical properties of auxetic cellular structures and resulted in improved performance for energy absorption applications.

#### 3.2.4. Summary/Manufacture of AMMs

Powder bed fusion methods are less prevalent in AMM research than that of vat polymerization; however, there is potential in the discussed methods (SLS, SLM, MJF and EBM) to contribute to the improved fabrication of AMMs. Namely, MJF shows significant capability in the fast production of polymer-based AMMs with improved mechanical properties and resolution on par with SLS. With further development, MJP could not only surpass other powder bed methods in mechanical part quality and minimum feature size but shows potential to exceed many vat polymerization and extrusion techniques as well. Below, the variables pertaining to the additive manufacture of AMMs for use in widespread industry are discussed, with the aforementioned techniques compared and contrasted for suitability in each category.

**Ability to mass-produce:**

MJF is the fastest production method presented in this sub-section, as it employs infrared projections to render an entire layer at once. This feature is not shared by the other powder bed fusion methods listed and is very useful for the batch production of AMM devices. The other methods employ a scanning regime for heating parts, meaning decreasing the layer thickness to achieve finer features will have a detrimental impact on the printing time. SLS and SLM examples are shown with printing speeds in the X–Y plane of 4000 mm/s and 1200 mm/s, respectively. An added benefit for AMM manufacture is these techniques require minimal support structures.

**Minimum printable feature size:**

The minimum feature sizes of all powder bed methods are roughly equivalent to each other, ranging from >400 μm (EBM) to 100–20 μm (SLM). The minimum feature size is heavily restricted by the particle size of the build powder, with the particle size always being smaller than the resulting layer thickness. Refining powder to nanoscale particle sizes would be expensive and not necessarily successful in increasing method resolution (due to other restrictive variables like laser intensity/focal diameter or scanning regime). Overall, powder bed methods are limited to microscale features—SLM presents the smallest feature size of 20 μm.

**Multi-material printing:**

In general, powder bed methods cannot yet create multi-material parts without additional processing/material addition after printing, with an example if this shown in Figure 32. Currently no method accounts for the cross contamination of powder that would occur, meaning the initial process can only use a single base powder. EBM has the strongest potential to be developed into a multi-material technique as it has the widest range of applicable materials, including both plastics and metals. However, EBM requires a vacuum-sealed build chamber to function, making it a costlier manufacturing process.

**Active metamaterial fabrication:**

SLS and MJF utilize plastics as the build material, which is generally more appropriate for fabricating standard AMMs, whereas SLM uses metal particles. Only SLM and EBM have metal-printing capabilities, which could be used to fabricate active metamaterials controlled by electromagnetic fields. Hydrogels are not compatible with any powder bed techniques, limiting the actuation possibilities of printed metamaterials.

### 3.3. Extrusion/Deposition Techniques

Extrusion-based techniques were among the first additive manufacturing techniques developed, with a significant literature surrounding this form of 3D printing in macro-scale. This technology is capable of multi-material and multicolour prints with a wide range of materials, such as plastics, ceramics [154], metals [155], food and even living cells [11]. Developing competitively smaller print sizes for these techniques is a newer concept, with the full potential of micro-extrusion not yet practically realized [156]. The techniques discussed below can be collectively described as layer-wise additive manufacturing methods, wherein deposited material builds a 3D part from the bottom-up. Down-scaling traditional extrusion methods, like fused deposition modelling (FDM), would have a broad range of industry applications, from biomedical devices to microelectronics and sensors. Additionally, micro-extrusion could provide greater design freedom, fewer processing steps, lower cost for small batch sizes and a variety of compatible build materials. This section also encompasses other deposition methods, with a focus on droplet-based based techniques, including direct ink writing (DIW) and jetting systems.

#### 3.3.1. Fused Deposition Modelling (FDM)

This method extrudes build material through a nozzle via a system using pneumatic or mechanical actuation [156]. The key difference for FDM is it uses a more continuous deposition of solid filament, whereas the other discussed additive methods are droplet-based (with liquid build material) and are therefore generally non-continuous. During its initial development in the 1990s, FDM primarily used thermoplastic polymer filament due to its low melt temperature, formability, and low price [11]. Nowadays, a variety of materials can be used, including through the addition of fibre inclusions, nanoparticles and other additives to improve the properties of the base material. A study into FDM printable materials used test specimens made with carbon composite filaments, along with their standard base materials (PLA and ABS). Their mechanical, rheological and thermal properties were measured and compared [157]. All composite samples showed improved mechanical properties from their pure base materials, with the tensile modulus of ABS samples increasing from 919.25 MPa to 2193.37 MPa, and from 1538.05 MPa to 2637.29 MPa for PLA samples, because of the addition of carbon fibres. Overall, FDM-printed parts with carbon fibre composite PLA performed the best mechanically.

**Figure 33:**
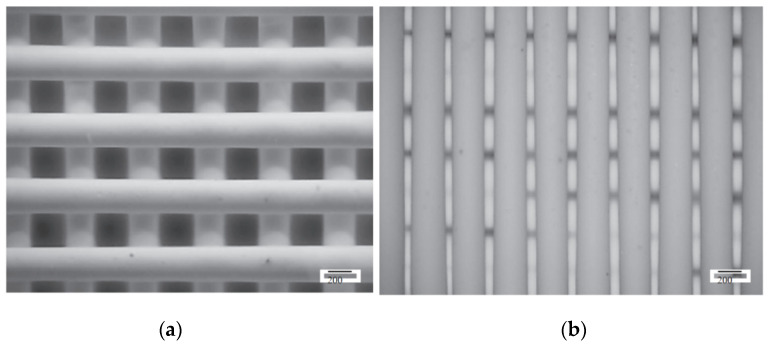
Microscopic images of zirconia lattices printed via freeform extrusion, with (**a**) 300 μm and (**b**) 70 μm size pores. Here, the scale bar is 200 μm [158].

**Figure 34:**
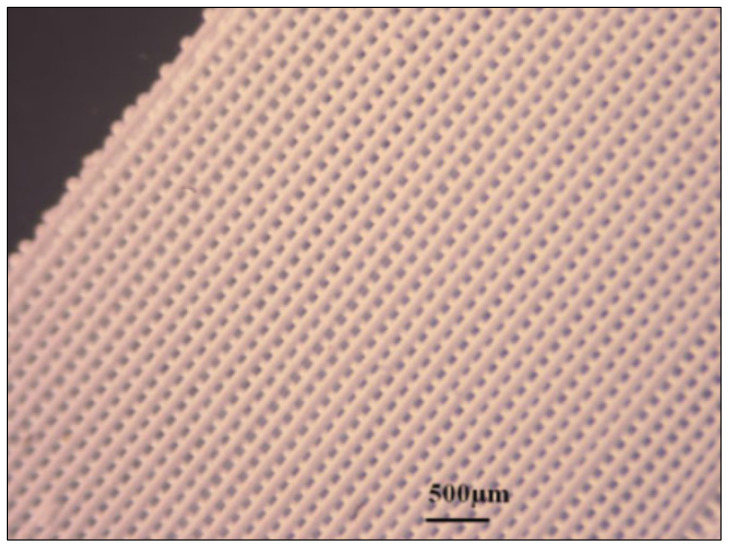
A fine ceramic (calcium phosphate) lattice printed via freeform extrusion and with a mesh spacing/pore size of 70 μm. Calcium phosphate is biocompatible and commonly used as bone substitution material. Here, the scale bar size is 500 μm. (Reprinted from [154], with the permission of John Wiley & Sons Inc).

A novel freeform extrusion technique derived from the principles of FDM has been used to fabricate high-density ceramic lattices [158]. The part was printed at low temperatures and achieved feature sizes well within the microscale, as shown in Figure 33. The lattice “woodpile” structure used is a well-known geometry in the AMM literature, and the acoustic response of the design was tested experimentally, confirming the band gap capabilities of the sonic lattice. Some defects/acoustic inaccuracies were present in the structure, likely due to microporosity and non-optimized printing parameters. In general, this solvent-based method proved efficient at producing microscale zirconia lattices, which could be applied in several potential AMM applications. Another freeform extrusion example produced fine ceramic lattices with spatial resolution lower than 100 μm, and it is shown in Figure 34 [154]. This method can be used with any ceramic powder, a feature that could be utilized in a range of AMM applications. The multi-size pores used in this design (from macro to submicron dimensions) and the biocompatible build materials are particularly useful for medical applications, such as drug delivery and hard tissue scaffolding. Fully realizing this extrusion technology would allow the rapid prototyping of 1–3 ceramic–polymer composites for piezoelectric bio-sensors.

**Figure 35:**
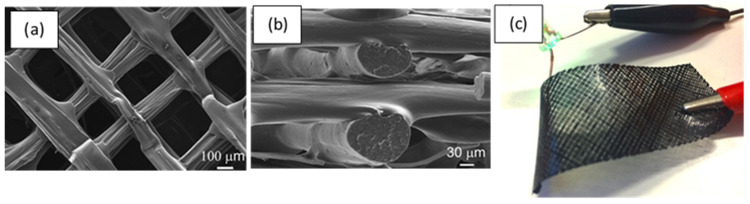
Carbon nanotube prototype with woven structure printed via liquid deposition modelling (a variant of FDM). The microstructure is displayed from (**a**) a top view and (**b**) a side view, with scale bars of 100 μm and 30 μm, respectively, and (**c**) shows the experimental confirmation of the prototypes electrical properties by connecting it to a simple electric circuit that lights up an LED [159]. (Reprinted from Composites Part A: Applied Science and Manufacturing, Volume 76, G. Postiglione, et al., ‘Conductive 3D microstructures by direct 3D printing of polymer/carbon nanotube nanocomposites via liquid deposition modeling’, Pages 110–114, Copyright (2015), with permission from Elsevier).

Another emerging FDM method involves the liquid deposition of a conductive nanocomposite, comprised of multiwall carbon nanotubes suspended in PLA, using a high volatility solvent as the dispersion medium [159]. This method not only has promising micro-fabrication abilities, but the printing setup requires only minor modifications to a commercially available, low-cost FDM printer to function. These modifications include adding a syringe dispenser to enable fluid deposition of the composite and solvent mixture. The solvent dispersion medium is important, as it ensures quick evaporation of the wet filament and therefore rapid construction of rigid microstructures. Printed features as small as 100 μm can be reproducibly obtained with the conductive nanocomposite, demonstrating the future potential of this novel technique to be developed into a viable micro-fabrication method for several key materials. A successfully-printed nanocarbon woven structure is shown in Figure 35. The prototype demonstrated its conductive capabilities by lighting up an LED in a simple electric circuit, as exhibited in Figure 35c, and has an average feature thickness of 100 μm, as shown in further detail by Figure 35a,b. With further development, this technology could allow for the cheap and reliable fabrication of microstructures in a variety of materials.

#### 3.3.2. Direct Ink Writing (DIW)

This method uses similar principles to FDM; however, the key difference is this method employs inks as the construction material [160]. Unlike FDM, time spent drying and solidifying the part is not necessary, as the shape retention comes from the rheological properties of the inks. This branch of additive manufacturing has extremely diverse material options, including ceramics, metal alloys, polymers, food, biomaterials, colloidal gels, hydrogels and electronically-functional matter. Additionally, DIW can construct complex shapes at low cost and without the use of masks or dies. DIW can be split into two definitive types: continuous filament/ink writing and inkjet printing. The former was originally developed as a technology for 2D image formation and has since progressed into a robust fabrication method wherein 3D parts are built up by the sequential selective deposition of ink droplets onto a substrate [2].

**Figure 36:**
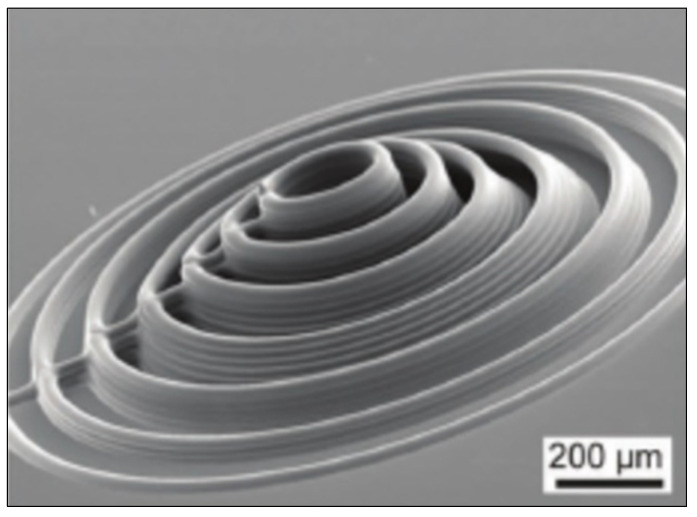
A metallic circular microstructure made using DIW with highly concentrated silver nanoparticle ink. The structure was fabricated layer-by-layer with continuous ink deposition and has a minimum feature size of 2 μm. Here, the scale bar is 200 μm. (Reprinted from [155], with the permission of John Wiley & Sons Inc.).

DIW is known to have a wide variety of ceramic and polymer material options, whereas current metallic material options are largely restricted to ink with silver particles [155]. Small-scale metallic parts can be DIW-formed by using concentrated metal nanoparticle inks with micrometre-sized (1–10 μm) nozzles. Figure 36 shows a metal microstructure printed using ink with a concentration of 75 wt% of 20 ± 5 nm silver nanoparticles. Metal-based DIW requires a post-printing annealing step to ensure structure stability. A novel modification to the DIW process countered this limitation by adding an in situ laser annealing system that anneals the ink as it leaves the nozzle. This alteration improved the minimum feature size of conventional DIW processes for metallic ink from 2 μm (for a 1 μm diameter nozzle) to as low as 600 nm. Additionally, conventional DIW has a printing rate of 20–500 μm/s, which increases to 0.5–1 mm/s once the laser annealing system is integrated. Simultaneous annealing and printing processes allow for almost arbitrary geometry to be created without the use of supports. This technology has been applied to make connecting wires for an array of micro-solar cells, demonstrating potential for various small-scale electronic applications.

**Figure 37:**
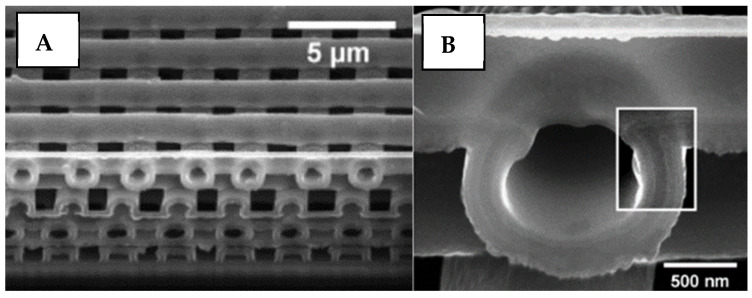
Magnification of hollow “woodpile” microstructure. The lattice design is made of silicon and formed using a direct ink written polymer scaffold that was then silicon-coated and melted to produce the hollow structure. The lattice is 8 by 8 with lateral dimensions of 250 μm by 250 μm. (**A**) Magnified image of lattice architecture with a scale bar of 5 μm. (**B**) Magnified image with a scale bar of 500 nm]. (Reprinted from [161], with the permission of John Wiley & Sons Inc.).

Typically, DIW techniques have a resolution strongly limited by their nozzle diameter; however, submicron feature sizes have been achieved using support structures and microcapillary nozzles of varying diameters [7]. A hollow silicon microlattice was constructed, as shown in Figure 37, using a DIW-printed polyelectrolyte ink scaffolding that enabled the structure to have a wall thickness of <0.5 μm [161]. The production method first requires a solid polymer lattice with a rod diameter of 1μm to be constructed via DIW (this does not represent the minimum feature size of DIW—rods with diameters as low as 600 nm can be made). Then, a thin (100 nm) silica coating is added, and the scaffold removed. The thermal properties of the polyelectrolyte ink mean it can be melted and subsequently removed at temperatures easily withstood by solid silica. Next a final silicon coating is added for a total wall thickness of roughly 300 nm. The spectral response and optical properties of the “woodpile” structure depends on the geometry of the original polymer lattice; as such, the dimensions can be tailored for the desired photonic effects. This method shows promise for the quick and simple fabrication of photonic crystals, as well as other novel devices like silicon-based microfluidic networks and low-cost MEMS.

**Figure 38:**
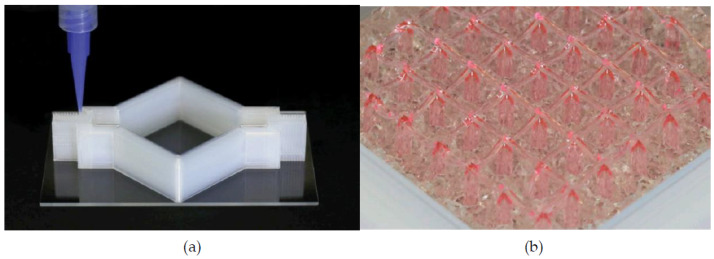
An DIW-fabricated vascularised tissue, with thickness exceeding 1 cm, printed in a 3D perfusion chip, showing (**a**) the chip that is printed in silicon and that enables perfusion within the tissue to occur for long time periods (greater than 6 weeks), and (**b**) the tissue, which is a multicellular matrix with embedded vascular structures (coloured red in the image). This tissue was constructed by co-printing several inks, which were composed of live cells (such as human stem cells and dermal fibroblasts) [162].

DIW technology can use a variety of ink formulations with different materials, including live cells and human tissue mimics. A novel printer set-up bio-printed thick (>1cm) vascularized tissues within 3D perfusion chips, as displayed in Figure 38 [162]. This complex fabrication involved the co-printing of several inks comprised of human stem cells to form an extracellular matrix with embedded vascular structures. Notably, this distinctive method can print tissues of arbitrary thickness, as the final product does not need to be UV cured. This means the method can be easily expanded to print other complex biomaterials.

DIW has been established as a high-resolution technique for a broad array of material options and with minimal cost [7]. Its liquid deposition regime coupled with the many available ink formulations are particularly useful to medical and bioprinting applications. Other breakthroughs in DIW technology include microfluidic multi-nozzle arrays, hydrogel and viscoelastic inks, printing soft-robotics, simultaneous multi-material printing and multi-core shell printing.

#### 3.3.3. Jetting Systems

Introduced in the previous DIW section, inkjet printing ejects ink onto a substrate using either controlled thermal or piezoelectric/mechanical actuation [2]. In the thermal regime, a printhead mounted above the nozzle sends electric pulses to heat the ejection site, which causes the ink to be expelled by local evaporation and air bubbles, whereas piezoelectric actuation involves the deformation of a piezocrystal, which pushes the ink volume through a nozzle. Thermal-based inkjet printing can propel droplets at pressures in excess of 125 atm and work with an operational efficiency of 160,000 droplets per second. The surface tension, inertia and viscosity of the ink must be carefully modulated to control the droplet deposition, as too high a viscosity can obstruct the fluid stream and too low a surface tension can cause nozzle leakage and affect printing resolution. Droplet size and nozzle diameter are the key parameters affecting the minimum print size for inkjet printing, which is typically below 10 μm but can be much smaller.

**Figure 39:**
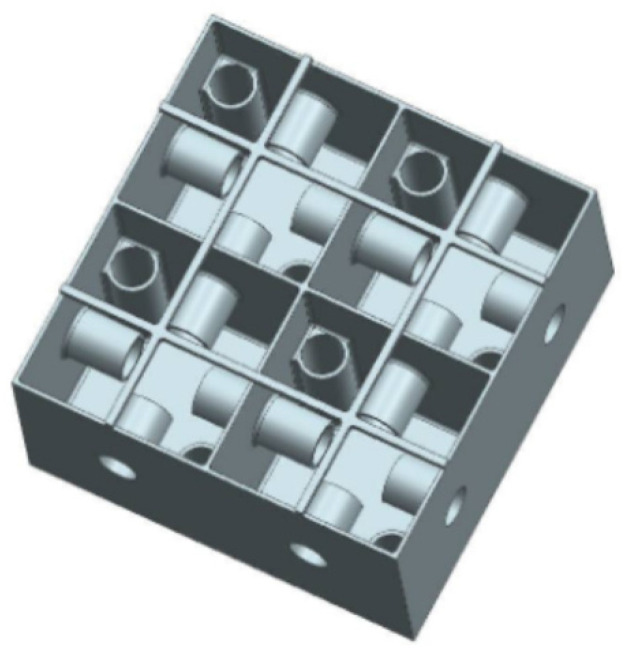
An AMM unit-cell for superior sound attenuation, fabricated using polyjet technology with polymer ink. The unit-cell is contained within a 50 mm wide cubic volume, with the printed prototype being 200 mm by 200 mm by 50 mm and containing 16 unit-cells [163]. (Reprinted from Procedia Engineering, Volume 176, R. Vdovin, et al., ‘Implementation of the Additive PolyJet Technology to the Development and Fabricating the Samples of the Acoustic Metamaterials’, Pages 595–599, Copyright (2017), with permission from Elsevier).

An application of interest for inkjet manufacturing methods is the production of acoustic metamaterial sound absorbers. An additive polyjet system was used to fabricate a thin-walled (1 mm thick), periodic AMM, with resonance exploited in the unit-cells by coupled tubes [163]. The geometry of the unit-cell, shown in Figure 39, is designed to achieve high sound absorption within a bandwidth of 300–600 Hz. Experimental testing of a printed prototype of 4 by 4 arranged unit-cells successfully demonstrated superior sound attenuation within the given frequency ranges, with twice the acoustic power absorbed compared to standard absorbers (such as wool-covered perforated panels of similar size). The polyjet setup has a dimensional accuracy of 20 μm in-plane and 16 μm out of plane and uses polymers as the construction medium, meaning the part requires a UV curing post-process.

**Figure 40:**
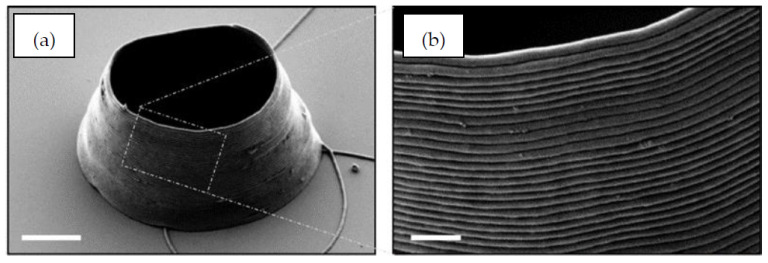
A cylindrical microstructure printed using EHD jetting with a novel electrostatic deflection system. The part was fabricated from polyethylene oxide (PEO) ink and achieved a submicron feature size without the use of supports. Here, a scale bar of (**a**) 5 μm and (**b**) 1 μm are used to image the microstructure [164].

An innovative manufacturing method uses precisely-controlled electrohydrodynamic (EHD) jets to achieve submicron feature sizes with extremely fast printing speeds [164]. Figure 40 shows a microscale part printed using this method, with a water-based ink containing polyethylene oxide (PEO) nanofibers. The EHD jets can reach speeds above 1 m/s, with ink propelled from the nozzle by a powerful applied electric field. By employing electrostatic deflection via controlled voltage input to several directing electrodes, the jet trajectory can be carefully modulated with lateral accelerations of up to 10^6^ m/s^2^. The layerwise regime of this method can stack up to 2000 sub-micrometre layers in one second, resulting in a printing speed three to four orders of magnitude faster than comparable methods with equivalent minimum feature sizes. An added benefit of electrohydrodynamic jetting is that nozzle clogging at the submicron scale is not an issue, unlike FDM, enabling nanometre-sized jets (less than 100 nm) to be used. The speed limits of this jetting technique are currently 0.5 m/s in-plane and 0.4 mm/s out of plane, and the resolution is currently limited to 100 nm (dependent on fibre thickness and jet apertures). This technology can be expanded to print with different ink compositions, including other polymers, biomaterials and living cells.

#### 3.3.4. Summary/Manufacture of AMMs

Extrusion/deposition methods are an extremely robust and versatile branch of additive manufacturing. This is reflected in its application in AMM manufacture (Figure 10, Figure 13 and Figure 39) and across several fields: from conductive nanocomposites (Figure 35) for use in MEMs, to tissue fabrication (Figure 38) for medical applications. Discussed in this section are FDM, DIW and jetting systems—notably, all these techniques excel at the range of printable materials available. In particular, ink-based methods (DIW and jetting systems) have an extremely diverse set of material options beyond the scope of most other printing techniques. Below, the variables pertaining to the additive manufacture of AMMs for use in widespread industry are discussed, with the aforementioned techniques compared and contrasted for suitability in each category.

**Ability to mass produce:**

FDM has the cheapest build material (solid filament) and likely the fastest production method due to the continuous material deposition regime. With DIW, small volumes of ink droplets are added discontinuously, leading to a slower print rate; however, jetting systems partially addresses this limitation. This is evident in the print rate of thermally-actuated jetting systems, which can be as high as 160,000 droplets per second. Some electrohydrodynamic setting systems can reach quick print rates of 0.5 m/s in-plane and 0.4 mm/s out of plane.

**Minimum printable feature size:**

The ink droplet-based methods offer extremely small printable feature capabilities, with resolutions of 2 μm to 600 nm for DIW and 10 μm to 100 nm for jetting systems. The minimum feature size has been greatly reduced by recent innovations in both these methods, such as integrated laser annealing for metallic DIW printing (Figure 36), and employing electrostatic jet deflection to direct ink placement (Figure 40). Aside from MPP, these methods offer the greatest resolution currently possible with additive manufacturing. In addition, FDM is by no means inadequate in terms of resolution—its minimum feature size of 100 μm puts its printing capabilities well into the microscale.

**Multi-material printing:**

In general, extrusion/deposition techniques have strong multi-material printing capabilities because instead of reacting with a large volume of build material, in a vat or bed, the part is built up using controlled addition of material. FDM has particularly strong multi-material printing capabilities because its solid filament has minimal risk of cross contamination and can easily be interchanged during printing. Ink-droplet methods also demonstrate these abilities, with a complex multi-material example presented in Figure 38. Coupled with the wide range of materials available (plastics, metals, ceramics, hydrogels, nanocarbons and live cells), these methods are very promising for the further production of multi-material parts.

**Active metamaterial fabrication:**

Although no active examples were provided in this section, the potential of these methods to achieve this is substantial. The printed examples consisting of ceramic (Figure 33 and Figure 34) or metallic (Figure 36) materials demonstrate the ability to fabricate metamaterials with piezoelectric or electromagnetic actuation. Carbon nanotube composites show particular promise for active metamaterials, as they are lightweight, flexible and electrically conductive. An example is the fabrication of a carbon nanotube microstructure, as seen in Figure 35, using a low-cost FDM printer with only minor modifications.

### 3.4. Hybrid Techniques

**Figure 41:**
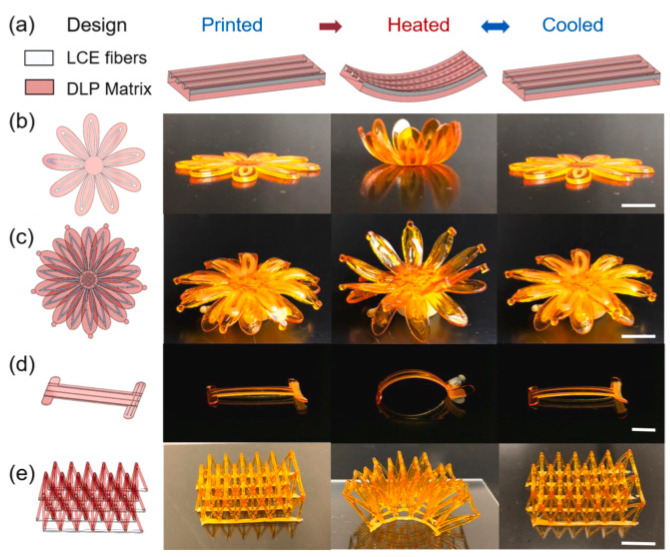
Several soft robot prototypes printed with a DLP–DIW hybrid method, with the four structure designs shown in (**b**–**e**) and a colour-coded key for the different materials in (**a**). These active structures are shown after printing (**left**), during heating/actuation (**middle**), and after cooling/relaxation (**right**). The structures are printed with layer thicknesses of 50 μm (for the DLP polymer matrix) and 700 μm (for the DIW LCE fibres), and the scale bar is 10 mm [165]. (Reprinted from Additive Manufacturing, Volume 40, X. Peng, et al., ‘Integrating digital light processing with direct ink writing for hybrid 3D printing of functional structures and devices’, Copyright (2021), with permission from Elsevier).

A novel hybrid technique combined digital light processing (DLP) and direct ink writing (DIW) to produce multi-material prints with functional inks, including liquid crystal elastomers (LCEs) and conductive silver inks [165]. This unique method combines both vat polymerization and ink deposition into one printing device capable of printing with both techniques simultaneously. The top-down DLP component had a resolution of 30–100 μm (dependent on focal length/frame adjustment), whereas the DIW syringe resolution range was from 100 μm to 1.54 mm (dependent on nozzle size used). This method shows promise for soft robotic applications, with examples presented using LCE fibres as the DIW print material and active component. Shown in Figure 41, this active structure consists of a DLP-printed soft elastomer matrix with the LCE fibres embedded using DIW, with layer thicknesses of 50 μm and 700 μm, respectively. The soft robots are reversibly actuated using temperature, with the fibres shrinking as a reaction to heat, hence causing the robot to bend/close. While this example is not in the microscopic range, the minimum layer thickness of 100 μm for this method shows promise for printing micron-size soft robots.

**Figure 42:**
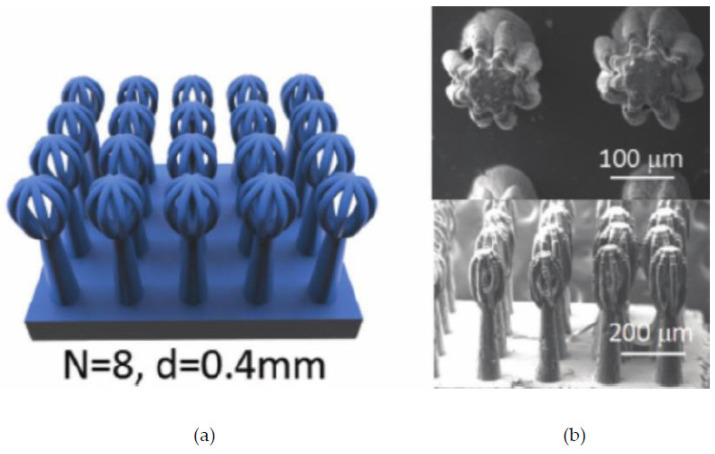
An example of a hydrophilic “egg-beater” microstructure printed using a DLP-based printing method, showing (**a**) the CAD model, and (**b**) an electron microscope image of the printed part, where the scale bar is 100 μm (**top**) and 200 μm (**bottom**). (Reprinted from [166], with the permission of John Wiley & Sons Inc.).

Another DLP-based process allows microscale features to be applied to macro-scale 3D objects, pre-printed with any suitable manufacturing method [166]. This process modifies the surface functionality of an inserted object to produce a hydrophobic effect. Figure 42 displays an example of the aforementioned hydrophobic microstructure, which can control the surface adhesion of water droplets by modulating the number of “egg beater” structures present.

While this example only demonstrates hydrophobic effects, a variety of surface properties could be induced with an appropriately designed microstructure. This DLP-based process can be used alongside virtually every additive manufacturing method and has the potential to revolutionize how surface effects are added to 3D-printed parts. Manipulating microdroplets in this fashion shows potential for use in several important applications, such as oil spill clean-up, micro reactors, 3D cell cultures and self-cleaning devices.

**Figure 43:**
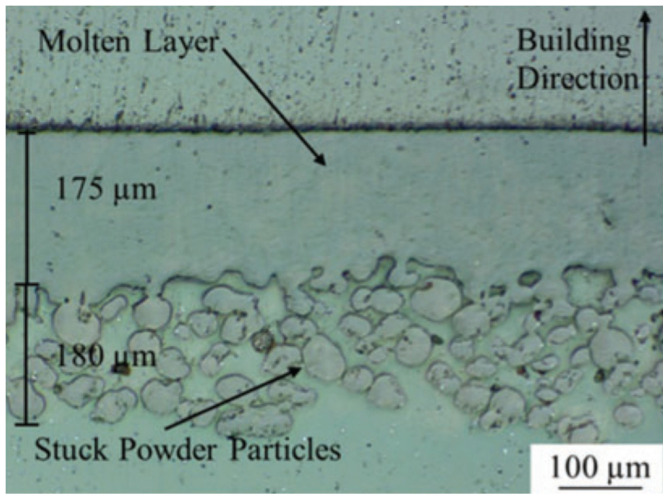
Cross-section of molten PA12 after 10 s simultaneous melting by a thulium laser, which is an integral step in the SLBM fabrication process. This study uses polypropylene (PP) and polyamide 12 (PA12) powder to print a multi-material part, where the resulting layer thickness is 175 μm. (Reprinted with permission from [167]. Copyright (2015), Laser Institute of America).

Adding DLP principles to the SLS/SLM manufacturing process has enabled simultaneous laser beam melting (SLBM) to be developed [167]. This technique allows several polymer powders to be melted and additively manufactured within one building process, therefore allowing the seamless production of multi-material parts. The process involves the polymer powders being deposited on the build platform and heated to just below melting temperature by infrared light emitters. Following this, CO_2_ and thulium lasers are used in conjunction with a DLP chip to achieve the simultaneous melting of both pre-heated polymers in the desired layer shape. Figure 43 displays a cross-section of the microstructure of a multi-material print after 10 s of thulium laser exposure, with a resulting layer thickness of 175 μm. Polypropylene (PP) and polyamide 12 (PA12) powders were used in Figure 43, which are both thermoplastic elastomers. SLBM presents a new approach to constructing multi-material polymer parts with microscale accuracy and short irradiation times; however, more research is required before it is a fully-realized additive manufacturing technique.

**Figure 44:**
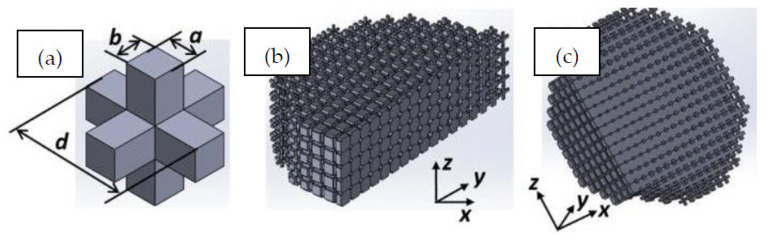
The geometry of the flattened Luneburg lens designs, fabricated using polyjet technology, showing (**a**) the periodic unit-cell of both lenses with dimensional variables appropriately labelled—this proposed building block can form a uniform 3D lattice once connected to adjacent, identical unit-cells; and (**b**) the 2D and (**c**) 3D lens designs printed with 5 and 19 stacked layers, respectively. (Reprinted from [168], with the permission of AIP Publishing).

A polyjet approach incorporating inkjet technology alongside the photocuring of resin, a distinctive feature of vat polymerization methods, has been applied to manufacture novel Luneburg lenses for the purposes of ultrasound beam steering [168]. The smallest feature of the meta-unit used in the lens, shown in Figure 44a, is 400 μm and designed to operate at an ultrasonic frequency of 40 kHz. The potential exists, however, to scale the meta-unit down to function at higher ultrasonic frequencies, because the polyjet printer used has a maximum possible resolution of 16 μm. The orthogonal truss meta-cells are interconnected to form a uniform 3D lattice that is both lightweight and self-supporting with minimal risk of deformation. The manufactured designs consist of a 2D and 3D flattened Luneburg lens, illustrated in Figure 44b,c, which are arranged in 5 and 19 stacked meta-cell layers, respectively. This fabrication technology offers a new avenue to fabricate ultrasonic beam-forming devices that is both efficient and precise in microscale resolutions. Polyjet printing is currently commercially available and already established as a robust printing method at marginally larger scales. As the demands for finer lattice geometry increase to surpass current bandwidth and frequency restrictions, polyjet printers show promise to maintain their continuous development to meet these ever-changing requirements. Should their consistent progress persevere, this technology demonstrates a clear feasibility in manufacturing AMMs for beam forming and other important industrial applications.

## 4. Discussion and Outlook

The field of acoustic metamaterials holds much promise for the future, with novel solutions to acoustic problems becoming increasingly close to real-world applications. While the field of AMMs covers such problems as earthquake engineering, building acoustics and underwater applications, we have chosen to focus on the potential uses of acoustic metamaterials on airborne systems for audio and ultrasonic applications.

With airborne sound covering wavelengths from metres to millimetres, the scale of materials that interact with these sounds is similar, so not only are novel subwavelength meta-atom designs important, but also the ability to create them is crucial. Additive manufacturing has proved to be the most promising out of existing technologies, exceeding its subtractive counterparts, to build up complex materials from their constituent meta-atoms. However, the outlook is challenging for translating this academic research into fully-realized devices for industrial and consumer applications. Rapid prototyping, while cheap for everyday use, still becomes expensive with scale, particularly techniques requiring custom materials. Additive technologies working at multiple scales is not feasible, so obtaining micron resolution over scales in excess of a centimetre is close to impossible. Complex shapes are challenging, and aspects of temperature dependence or curing/annealing mechanisms of raw materials can mean that it is difficult to print with adequate accuracy and reliability. For example, the printing of membranes—commonly used to provide mechanical behaviour to a meta-atom—is challenging, where at a small scale the raw materials can have post-production tension and warping that is difficult to compensate for. In addition, each technique has production requirements unique to itself. As mentioned, vat polymerization techniques cannot print voids or inclusions directly, instead requiring vents or other ways to leak out the original liquid polymer. Designs of AMMs need to account for that, providing another barrier to production.

Clearly, for a practical and realisable AMMs, we need to be able to create large-scale materials (and ultimately devices) with small-scale features. Additive manufacturing is the best candidate for both rapid prototyping and bulk manufacture, but limitations on specific fabrication characteristics act as a barrier to AMM research, constraining printable ideas and restricting its potential. Given that metamaterials typically derive their properties from repeating units of local resonators or geometrical features, a key issue in their manufacture is consistency and reproducibility across length scales. A material in which some of the meta-atoms failed to print correctly could alter the acoustic properties and potentially disrupt the predicted acoustic behaviour. This high dependency on uniformity, or reliability of shape, across meta-atoms means that AMMs are still very challenging to create, even with additive manufacturing, and especially at smaller scales. The robustness of metamaterials to meta-atom failure could be a key area of research to progress AMMs to commonplace use.

This review discussed many viable additive manufacturing methods for small structures, and it discussed techniques best suited to address some key fabrication parameters for AMMs. MPP was found to have the smallest printable feature size, followed by DIW, jetting systems and DLP, with the highest resolution of these methods well within the nanoscale. This result is supported by similar findings in the literature [113,169]. For structures requiring varied material options, like active AMMs, DIW and jetting systems were found to have the widest material range—from ceramics and metals to live cells—and a minimal risk of cross-contamination of materials, unlike vat polymerisation and powder bed methods. Additionally, extrusion/deposition methods are regarded as comparatively fast additive manufacture techniques, further demonstrating the significant potential of DIW and jetting systems as extremely effective and well-rounded techniques for AMM manufacture.

Other noteworthy techniques include SLM and EBM, which excel at fabricating metallic parts with microscale resolution without the use of support structures. Vat polymerisation and ink deposition methods are the set of techniques best suited to printing hydrogels—a key material for the fabrication of some active metamaterial designs. Vat polymerisation shows strong potential for AMM fabrication, with SLA, DLP and MPP all being robust techniques with individual benefits; however, their main limitation lies in their material restrictions and post-processing requirements. Further development to overcome these challenges would be beneficial for their application in AMM construction.

Over the following 10 years, the next generation of practical AMMs will need to come from either significant progress in rapid prototyping and manufacturing technology, or the discovery of meta-atom designs that have complex physical behaviour while being sufficiently simple enough to be printed. Active acoustic metamaterials promise to push development forward, compensating for manufacturing challenges by adding complexity through controlled actuation rather than static structure, but they are also difficult to produce beyond very basic systems at the smallest scale. Nevertheless, it will be exciting to monitor which of these two strands of research will succeed first.

## References

[1] Olsson Iii R.H., El-Kady I. (2008). Microfabricated phononic crystal devices and applications. Meas. Sci. Technol..

[2] Fritzler K.B., Prinz V.Y. (2019). 3D printing methods for micro- and nanostructures. Phys. Uspekhi.

[3] Lee D., Nguyen D.M., Rho J. (2017). Acoustic wave science realized by metamaterials. Nano Converg..

[4] Panda D., Mohanty A.R., Singh M., Rafat Y. (2021). Sonic crystals for highway noise reduction. Recent Developments in Acoustics.

[5] Acoustic Metamaterials Group Acousticmetamaterials.org. https://acousticmetamaterials.org/.

[6] MetAcoustic Metacoustic.com. https://metacoustic.com/.

[7] Mao M., Mao M., He J., Li X., Zhang B., Lei Q., Liu Y., Li D. (2017). The emerging frontiers and applications of high-resolution 3D printing. Micromachines.

[8] Kjar A., Huang Y. (2019). Application of micro-scale 3D printing in pharmaceutics. Pharmaceutics.

[9] Ahn S.-H., Yoon H.-S., Jang K.-H., Kim E.-S., Lee H.-T., Lee G.-Y., Kim C.-S., Cha S.-W. (2015). Nanoscale 3D printing process using aerodynamically focused nanoparticle (AFN) printing, micro-machining, and focused ion beam (FIB). CIRP Ann..

[10] Liu Z., Zhan J., Fard M., Davy J.L. (2017). Acoustic measurement of a 3D printed micro-perforated panel combined with a porous material. Measurement.

[11] Shahrubudin N., Lee T.C., Ramlan R. (2019). An overview on 3D printing technology: Technological, materials, and applications. Procedia Manuf..

[12] Savini A., Savini G.G. A short history of 3D printing, a technological revolution just started. Proceedings of the 2015 ICOHTEC/IEEE International History of High-Technologies and their Socio-Cultural Contexts Conference (HISTELCON).

[13] Paoletti I., Ceccon L. (2018). The evolution of 3D printing in AEC: From experimental to consolidated techniques. 3D Printing.

[14] Noorani R. (2017). 3D Printing: Technology, Applications, and Selection.

[15] Goral J., Deo M. (2020). Nanofabrication of synthetic nanoporous geomaterials: From nanoscale-resolution 3D imaging to nano-3D-printed digital (shale) rock. Sci. Rep..

[16] Audoly C. (2019). Acoustic metamaterials and underwater acoustics applications. Fundam. Appl. Acoust. Metamaterials.

[17] Laureti S. (2016). Acoustic Metamaterials for Medical Ultrasound and Non-Destructive Evaluation. Ph.D. Thesis.

[18] Kan W., Liang B., Li R., Jiang X., Zou X.-Y., Yin L.-L., Cheng J. (2016). Three-dimensional broadband acoustic illusion cloak for sound-hard boundaries of curved geometry. Sci. Rep..

[19] Wu J.J., Ma F., Zhang S., Shen L. (2016). Application of acoustic metamaterials in low-frequency vibration and noise reduction. J. Mech. Eng..

[20] Ma G., Sheng P. (2016). Acoustic metamaterials: From local resonances to broad horizons. Sci. Adv..

[21] Fok L., Ambati M., Zhang X. (2008). Acoustic metamaterials. MRS Bull..

[22] Zangeneh-Nejad F., Fleury R. (2019). Active times for acoustic metamaterials. Rev. Phys..

[23] Gan W.S. (2012). New acoustics based on metamaterials. Acoustical Imaging: Techniques and Applications for Engineers.

[24] Craster R.V., Guenneau S. (2013). Chapter 1: Fundamentals of acoustic metamaterials. Acoustic Metamaterials: Negative Refraction, Imaging, Lensing and Cloaking.

[25] Alster M. (1972). Improved calculation of resonant frequencies of helmholtz resonators. J. Sound Vib..

[26] Dalela S., Balaji P.S., Jena D.P. (2021). A review on application of mechanical metamaterials for vibration control. Mech. Adv. Mater. Struct..

[27] Akl W., Baz A. (2013). Active acoustic metamaterial with simultaneously programmable density and bulk modulus. J. Vib. Acoust..

[28] Akl W., Baz A. (2010). Multi-cell active acoustic metamaterial with programmable bulk modulus. J. Intell. Mater. Syst. Struct..

[29] Xia B., Chen N., Xie L., Qin Y., Yu D. (2016). Temperature-controlled tunable acoustic metamaterial with active band gap and negative bulk modulus. Appl. Acoust..

[30] Baz A. (2009). The structure of an active acoustic metamaterial with tunable effective density. New J. Phys..

[31] Baz A.M. (2010). An active acoustic metamaterial with tunable effective density. J. Vib. Acoust..

[32] Akl W., Baz A. (2012). Experimental characterization of active acoustic metamaterial cell with controllable dynamic density. J. Appl. Phys..

[33] Chen Z., Xue C., Fan L., Zhang S.-Y., Li X.-J., Zhang H., Ding J. (2016). A tunable acoustic metamaterial with double-negativity driven by electromagnets. Sci. Rep..

[34] Kumar S., Lee H.P. (2019). Recent advances in active acoustic metamaterials. Int. J. Appl. Mech..

[35] Chen S., Fan Y., Fu Q., Wu H., Jin Y., Zheng J., Zhang F. (2018). A review of tunable acoustic metamaterials. Appl. Sci..

[36] Zhang H., Guo X., Wu J., Fang D., Zhang Y. (2018). Soft mechanical metamaterials with unusual swelling behavior and tunable stress-strain curves. Sci. Adv..

[37] Baz A. (2019). Active acoustic metamaterial with tunable effective density using a disturbance rejection controller. J. Appl. Phys..

[38] Akl W., Baz A. (2012). Analysis and experimental demonstration of an active acoustic metamaterial cell. J. Appl. Phys..

[39] Kumar S., Lee H.P. (2020). Recent advances in acoustic metamaterials for simultaneous sound attenuation and air ventilation performances. Crystals.

[40] Nejad F.Z., Fleury R. (2018). Potential applications of high-index acoustic metamaterials. J. Acoust. Soc. Am..

[41] Fedotov V., Kasap S., Capper P. (2017). Metamaterials. Springer Handbook of Electronic and Photonic Materials.

[42] Veselago V.G. (1968). The electrodynamics of substances with simultaneously negative values of ϵ and μ. Sov. Phys. Uspekhi.

[43] Pendry J.B., Holden A.J., Stewart W.J., Youngs I. (1996). Extremely low frequency plasmons in metallic mesostructures. Phys. Rev. Lett..

[44] Sigalas M., Economou E. (1993). Band structure of elastic waves in two dimensional systems. Solid State Commun..

[45] Espinosa F.R.M.D., Jimeniz E., Torres M. Experimental assessment of an ultrasonic band gap in a periodic two-dimensional composite. Proceedings of the 1997 IEEE Ultrasonics Symposium Proceedings. An International Symposium (Cat. No.97CH36118).

[46] Sugimoto N. (1992). Propagation of nonlinear acoustic waves in a tunnel with an array of helmholtz resonators. J. Fluid Mech..

[47] Khelif A., Adibi A. (2015). Phononic Crystals: Fundamentals and Applications.

[48] Wang G., Yu D., Wen J., Liu Y., Wen X. (2004). One-dimensional phononic crystals with locally resonant structures. Phys. Lett. A.

[49] Pennec Y., Vasseur J.O., Djafari-Rouhani B., Dobrzyński L., Deymier P.A. (2010). Two-dimensional phononic crystals: Examples and applications. Surf. Sci. Rep..

[50] Khelif A., Hsiao F.-L., Choujaa A., Benchabane S., Laude V. (2010). Octave omnidirectional band gap in a three-dimensional phononic crystal. IEEE Trans. Ultrason. Ferroelectr. Freq. Control.

[51] Armenise M.N., Campanella C.E., Ciminelli C., Dell’Olio F., Passaro V.M. (2010). Phononic and photonic band gap structures: Modelling and applications. Phys. Procedia.

[52] Olsson R.H., Griego S.X., El-Kady I., Su M., Soliman Y., Goettler D., Leseman Z. Ultra high frequency (UHF) phononic crystal devices operating in mobile communication bands. Proceedings of the 2009 IEEE International Ultrasonics Symposium.

[53] Lucklum R. (2014). Phononic crystals and metamaterials—Promising new sensor platforms. Procedia Eng..

[54] Torrent D., Sánchez-Dehesa J. (2008). Acoustic cloaking in two dimensions: A feasible approach. New J. Phys..

[55] Cummer S., Schurig D. (2007). One path to acoustic cloaking. New J. Phys..

[56] Zhang S., Xia C., Fang N. (2011). Broadband acoustic cloak for ultrasound waves. Phys. Rev. Lett..

[57] Zigoneanu L., Popa B.-I., Cummer S.A. (2014). Three-dimensional broadband omnidirectional acoustic ground cloak. Nat. Mater..

[58] Zhu J., Chen T., Song X., Chen C., Liu Z., Zhang J. (2019). Three-dimensional large-scale acoustic invisibility cloak with layered metamaterials for underwater operation. Phys. Scr..

[59] Elford D.P., Chalmers L., Kusmartsev F.V., Swallowe G.M. (2011). Matryoshka locally resonant sonic crystal. J. Acoust. Soc. Am..

[60] Pennec Y., Djafari-Rouhani B., Khelif A., Adibi A. (2016). Fundamental properties of phononic crystal. Phononic Crystals: Fundamentals and Applications.

[61] Krushynska A., Miniaci M., Bosia F., Pugno N. (2017). Coupling local resonance with bragg band gaps in single-phase mechanical metamaterials. Extrem. Mech. Lett..

[62] Lu M.-H., Feng L., Chen Y.-F. (2009). Phononic crystals and acoustic metamaterials. Mater. Today.

[63] Kushwaha M.S., Halevi P. (1996). Giant acoustic stop bands in two-dimensional periodic arrays of liquid cylinders. Appl. Phys. Lett..

[64] Kushwaha M.S., Djafari-Rouhani B. (1998). Giant sonic stop bands in two-dimensional periodic system of fluids. J. Appl. Phys..

[65] Sigalas M.M. (1998). Defect states of acoustic waves in a two-dimensional lattice of solid cylinders. J. Appl. Phys..

[66] Li J., Chan C.T. (2004). Double-negative acoustic metamaterial. Phys. Rev. E Stat. Nonlinear Soft Matter Phys..

[67] Liu Z., Zhang X., Mao Y., Zhu Y.Y., Yang Z., Chan C.T., Sheng P. (2000). Locally resonant sonic materials. Science.

[68] Jena D., Dandsena J., Jayakumari V. (2019). Demonstration of effective acoustic properties of different configurations of helmholtz resonators. Appl. Acoust..

[69] Fang N., Xi D., Xu J., Ambati M., Srituravanich W., Sun C., Zhang X. (2006). Ultrasonic metamaterials with negative modulus. Nat. Mater..

[70] Kim S.-H., Lee S.-H. (2014). Air transparent soundproof window. AIP Adv..

[71] Kumar S., Bhushan P., Prakash O., Bhattacharya S. (2018). Double negative acoustic metastructure for attenuation of acoustic emissions. Appl. Phys. Lett..

[72] Lee S.H., Park C.M., Seo Y.M., Wang Z.G., Kim C.K. (2010). Composite acoustic medium with simultaneously negative density and modulus. Phys. Rev. Lett..

[73] Xu Y., Wu J.H., Cai Y., Ma F. (2019). Investigation on dynamic effective parameters of perforated thin-plate acoustic metamaterials. J. Phys. D Appl. Phys..

[74] Tan K.T., Huang H.H., Sun C.T. (2012). Optimizing the band gap of effective mass negativity in acoustic metamaterials. Appl. Phys. Lett..

[75] Su H., Zhou X., Xu X., Hu G. (2014). Experimental study on acoustic subwavelength imaging of holey-structured metamaterials by resonant tunneling. J. Acoust. Soc. Am..

[76] Chen Y., Zhu R., Nguyen H.Q., Huang G., Pai P., Huang G. (2015). Chapter 3 Membrane-type acoustic metamaterials: Theory, design, and application. Theory and Design of Acoustic Metamaterials.

[77] Huang T.-Y., Shen C., Jing Y. (2016). Membrane- and plate-type acoustic metamaterials. J. Acoust. Soc. Am..

[78] Naify C.J., Chang C.-M., McKnight G., Nutt S.R. (2011). Transmission loss of membrane-type acoustic metamaterials with coaxial ring masses. J. Appl. Phys..

[79] Lu Z., Yu X., Lau S.-K., Khoo B.C., Cui F. (2020). Membrane-type acoustic metamaterial with eccentric masses for broadband sound isolation. Appl. Acoust..

[80] Naify C.J., Chang C.-M., McKnight G., Nutt S.R. (2012). Scaling of membrane-type locally resonant acoustic metamaterial arrays. J. Acoust. Soc. Am..

[81] Casarini C., Romero-Garcia V., Groby J.-P., Tiller B., Windmill J.F.C., Jackson J.C., Tiller B. (2019). Fabrication and characterization of 3D printed thin plates for acoustic metamaterials applications. IEEE Sens. J..

[82] Li Y., Liang B., Gu Z.-M., Zou X.-Y., Cheng J.-C. (2013). Reflected wavefront manipulation based on ultrathin planar acoustic metasurfaces. Sci. Rep..

[83] Guo J., Zhang X., Fang Y., Fattah R. (2018). Manipulating reflected acoustic wave via Helmholtz resonators with varying-length extended necks. J. Appl. Phys..

[84] Liang Z., Feng T., Lok S., Liu F., Ng K.B., Chan C.H., Wang J., Han S., Lee S., Li J. (2013). Space-coiling metamaterials with double negativity and conical dispersion. Sci. Rep..

[85] Maurya S.K., Pandey A., Shukla S., Saxena S. (2016). Double negativity in 3D space coiling metamaterials. Sci. Rep..

[86] Chen T., Jiao J., Yu D. (2020). Enhanced broadband acoustic sensing in gradient coiling up metamaterials. J. Phys. D Appl. Phys..

[87] Song G.Y., Cheng Q., Huang B., Dong H.Y., Cui T.J. (2016). Broadband fractal acoustic metamaterials for low-frequency sound attenuation. Appl. Phys. Lett..

[88] Akl W., Baz A. (2021). Active control of the dynamic density of acoustic metamaterials. Appl. Acoust..

[89] Baz A. (2020). Active synthesis of a gyroscopic-nonreciprocal acoustic metamaterial. J. Acoust. Soc. Am..

[90] Shen H., Païdoussis M.P., Wen J., Yu D., Cai L., Wen X. (2012). Acoustic cloak/anti-cloak device with realizable passive/active metamaterials. J. Phys. D Appl. Phys..

[91] Bacigalupo A., de Bellis M.L., Misseroni D. (2019). Design of Active Acoustic Metamaterials with Periodic Piezoelectric Microstructure. arXiv Prepr..

[92] Hedayati R., Lakshmanan S. (2020). Pneumatically-actuated acoustic metamaterials based on helmholtz resonators. Materials.

[93] Zhang K., Ma C., He Q., Lin S., Chen Y., Zhang Y., Fang N.X., Zhao X. (2019). Metagel with broadband tunable acoustic properties over air–water–solid ranges. Adv. Funct. Mater..

[94] Fraternali F., Senatore L., Daraio C. (2012). Solitary waves on tensegrity lattices. J. Mech. Phys. Solids.

[95] Fraternali F., Carpentieri G., Amendola A., Skelton R.E., Nesterenko V.F. (2014). Multiscale tunability of solitary wave dynamics in tensegrity metamaterials. Appl. Phys. Lett..

[96] Spadoni A., Daraio C. (2010). Generation and control of sound bullets with a nonlinear acoustic lens. Proc. Natl. Acad. Sci. USA.

[97] Vangelatos Z., Micheletti A., Grigoropoulos C.P., Fraternali F. (2020). Design and testing of bistable lattices with tensegrity architecture and nanoscale features fabricated by multiphoton lithography. Nanomaterials.

[98] Skelton R.E., de Oliveira M.C. (2009). Tensegrity Systems.

[99] Akl W., Baz A. (2011). Stability analysis of active acoustic metamaterial with programmable bulk modulus. Smart Mater. Struct..

[100] Liang Z., Willatzen M., Li J., Christensen J. (2012). Tunable acoustic double negativity metamaterial. Sci. Rep..

[101] Ning L., Wang Y.-Z., Wang Y.-S. (2019). Active control of elastic metamaterials consisting of symmetric double helmholtz resonator cavities. Int. J. Mech. Sci..

[102] Muguruza A., Bo J.B., Gómez A., Minguella-Canela J., Fernandes J., Ramos F., Xuriguera E., Varea A., Cirera A. (2017). Development of a multi-material additive manufacturing process for electronic devices. Procedia Manuf..

[103] Zhao Z., Tian X., Song X. (2020). Engineering materials with light: Recent progress in digital light processing based 3D printing. J. Mater. Chem. C.

[104] Cvetkovic C., Raman R., Chan V., Williams B.J., Tolish M., Bajaj P., Sakar M.S., Asada H.H., Saif M.T.A., Bashir R. (2014). Three-dimensionally printed biological machines powered by skeletal muscle. Proc. Natl. Acad. Sci. USA.

[105] Bagheri A., Jin J. (2019). Photopolymerization in 3D printing. ACS Appl. Polym. Mater..

[106] Uzcategui A.C., Muralidharan A., Ferguson V.L., Bryant S.J., McLeod R.R. (2018). Understanding and improving mechanical properties in 3D printed parts using a dual-cure acrylate-based resin for stereolithography. Adv. Eng. Mater..

[107] Fleming A.J., Ghalehbeygi O.T., Routley B.S., Wills A.G. (2017). Experimental scanning laser lithography with exposure optimization. IFAC-PapersOnLine.

[108] Casarini C., Windmill J.F.C., Jackson J.C. 3D printed small-scale acoustic metamaterials based on Helmholtz resonators with tuned overtones. Proceedings of the 2017 IEEE Sensors.

[109] Asiga.com. Asiga Products—MAX X. https://www.asiga.com/products/printers/max_series/max_x/.

[110] Daly P., Windmill J.F.C., Jackson J., Vasilev M. An in-air ultrasonic acoustic beam shifter metamaterial. Proceedings of the IEEE IUS Symposium.

[111] Maruyama T., Hirata H., Furukawa T., Maruo S. (2020). Multi-material microstereolithography using a palette with multicolor photocurable resins. Opt. Mater. Express.

[112] Hu Y., Guo Z., Ragonese A., Zhu T., Khuje S., Li C., Grossman J.C., Zhou C., Nouh M., Ren S. (2020). A 3D-printed molecular ferroelectric metamaterial. Proc. Natl. Acad. Sci. USA.

[113] Ge Q., Li Z., Wang Z., Kowsari K., Zhang W., He X., Zhou J., Fang N.X. (2020). Projection micro stereolithography based 3D printing and its applications. Int. J. Extrem. Manuf..

[114] Ertugrul I. (2020). The fabrication of micro beam from photopolymer by digital light processing 3D printing technology. Micromachines.

[115] Tumbleston J.R., Shirvanyants D., Ermoshkin N., Janusziewicz R., Johnson A.R., Kelly D., Chen K., Pinschmidt R., Rolland J.P., Ermoshkin A. (2015). Continuous liquid interface production of 3D objects. Science.

[116] Zhou C., Chen Y., Yang Z., Khoshnevis B. Development of a multi-material mask-image-projection-based stereolithography for the fabrication of digital materials. Proceedings of the 22nd Annual International Solid Freeform Fabrication Symposium—An Additive Manufacturing Conference.

[117] Kowsari K., Akbari S., Wang D., Fang N.X., Ge Q. (2018). High-efficiency high-resolution multimaterial fabrication for digital light processing-based three-dimensional printing. 3D Print. Addit. Manuf..

[118] Han D., Yang C., Fang N.X., Lee H. (2019). Rapid multi-material 3D printing with projection micro-stereolithography using dynamic fluidic control. Addit. Manuf..

[119] Han D., Lu Z., Chester S.A., Lee H. (2018). Micro 3D printing of a temperature-responsive hydrogel using projection micro-stereolithography. Sci. Rep..

[120] Schwartz J.J., Boydston A.J. (2019). Multimaterial actinic spatial control 3D and 4D printing. Nat. Commun..

[121] Khatri B., Frey M., Raouf-Fahmy A., Scharla M.-V., Hanemann T. (2020). Development of a multi-material stereolithography 3D printing device. Micromachines.

[122] Loterie D., Delrot P., Moser C. (2020). High-resolution tomographic volumetric additive manufacturing. Nat. Commun..

[123] Maruo S., Nakamura O., Kawata S. (1997). Three-dimensional microfabrication with two-photon-absorbed photopolymerization. Opt. Lett..

[124] Yahya B., El I.Y., Imad M., Zouheir S. (2019). Direct laser writing of submicrometric voxels in two-photon photopolymerization. Proceedings SPIE 11098, Molecular and Nano Machines II.

[125] Huang Z., Tsui G.C.-P., Deng Y., Tang C.-Y. (2020). Two-photon polymerization nanolithography technology for fabrication of stimulus-responsive micro/nano-structures for biomedical applications. Nanotechnol. Rev..

[126] Haske W., Chen V.W., Hales J.M., Dong W., Barlow S., Marder S.R., Perry J.W. (2007). 65 Nm feature sizes using visible wavelength 3-D multiphoton lithography. Opt. Express.

[127] Tan D., Li Y., Qi F., Yang H., Gong Q., Dong X., Duan X. (2007). Reduction in feature size of two-photon polymerization using SCR500. Appl. Phys. Lett..

[128] Ovsianikov A., Viertl J., Chichkov B., Oubaha M., MacCraith B., Sakellari I., Giakoumaki A., Gray D., Vamvakaki M., Farsari M. (2008). Ultra-low shrinkage hybrid photosensitive material for two-photon polymerization microfabrication. ACS Nano.

[129] Yeung K.-W., Dong Y., Chen L., Tang C.-Y., Law W.-C., Tsui G.C.-P., Engstrøm D.S. (2020). Printability of photo-sensitive nanocomposites using two-photon polymerization. Nanotechnol. Rev..

[130] Nguyen A.K., Narayan R.J. (2017). Two-photon polymerization for biological applications. Mater. Today.

[131] Gottmann J., Hoerstmann-Jungemann M., Hermans M., Beckmann D. (2009). High speed and high precision fs-laser writing using a scanner with large numerical aperture. JLMN.

[132] Pearre B.W., Michas C., Tsang J.-M., Gardner T.J., Otchy T.M. (2019). Fast micron-scale 3D printing with a resonant-scanning two-photon microscope. Addit. Manuf..

[133] Carlotti M., Mattoli V. (2019). Functional materials for two-photon polymerization in microfabrication. Small.

[134] Tudor A., Delaney C., Zhang H., Thompson A.J., Curto V.F., Yang G.-Z., Higgins M.J., Diamond D., Florea L. (2018). Fabrication of soft, stimulus-responsive structures with sub-micron resolution via two-photon polymerization of poly(ionic liquid)s. Mater. Today.

[135] Xing J.-F., Zheng M.-L., Duan X.-M. (2015). Two-photon polymerization microfabrication of hydrogels: An advanced 3D printing technology for tissue engineering and drug delivery. Chem. Soc. Rev..

[136] Weisgrab G., Guillaume O., Guo Z., Heimel P., Slezak P., Poot A., Grijpma D., Ovsianikov A. (2020). 3D printing of large-scale and highly porous biodegradable tissue engineering scaffolds from poly(trimethylene-carbonate) using two-photon-polymerization. Biofabrication.

[137] Raimondi M.T., Eaton S.M., Nava M.M., Laganà M., Cerullo G., Osellame R. (2012). Two-photon laser polymerization: From fundamentals to biomedical application in tissue engineering and regenerative medicine. J. Appl. Biomater. Funct. Mater..

[138] Amato L., Gu Y., Bellini N., Eaton S.M., Cerullo G., Osellame R. (2012). Integrated three-dimensional filter separates nanoscale from microscale elements in a microfluidic chip. Lab Chip.

[139] Singh R., Gupta A., Tripathi O., Srivastava S., Singh B., Awasthi A., Rajput S., Sonia P., Singhal P., Saxena K.K. (2020). Powder bed fusion process in additive manufacturing: An overview. Mater. Today Proc..

[140] Yang W., Bai X., Zhu W., Kiran R., An J., Chua C.K., Zhou K. (2020). 3D printing of polymeric multi-layer micro-perforated panels for tunable wideband sound absorption. Polymers.

[141] Claeys C., Deckers E., Pluymers B., Desmet W. (2016). A Lightweight vibro-acoustic metamaterial demonstrator: Numerical and experimental investigation. Mech. Syst. Signal Process..

[142] Yuan S., Shen F., Bai J., Chua C.K., Wei J., Zhou K. (2017). 3D soft auxetic lattice structures fabricated by selective laser sintering: TPU powder evaluation and process optimization. Mater. Des..

[143] Nagarajan B., Hu Z., Song X., Zhai W., Wei J. (2019). Development of micro selective laser melting: The state of the art and future perspectives. Engineering.

[144] Fischer J., Kniepkamp M., Abele E. Micro laser melting: Analyses of current potentials and restrictions for the additive manufacturing of micro structures. Proceedings of the 25th Annual International Solid Freeform Fabrication Symposium—An additive Manufacturing Conference.

[145] Gieseke M., Senz V., Vehse M., Fiedler S., Irsig R., Hustedt M., Sternberg K., Nölke C., Kaierle S., Wesling V. (2012). Additive manufacturing of drug delivery systems. Biomed. Eng. Biomed. Tech..

[146] Wang Y., Ren X., Chen Z., Jiang Y., Cao X., Fang S., Zhao T., Li Y., Fang D. (2020). Numerical and experimental studies on compressive behavior of gyroid lattice cylindrical shells. Mater. Des..

[147] Cao X., Jiang Y., Zhao T., Wang P., Wang Y., Chen Z., Li Y., Xiao D., Fang D. (2020). Compression experiment and numerical evaluation on mechanical responses of the lattice structures with stochastic geometric defects originated from additive-manufacturing. Compos. Part B Eng..

[148] Zhang L., Song B., Fu J., Wei S., Yang L., Yan C., Li H., Gao L., Shi Y. (2020). Topology-optimized lattice structures with simultaneously high stiffness and light weight fabricated by selective laser melting: Design, manufacturing and characterization. J. Manuf. Process..

[149] He J., Jiang X., Ta D., Wang W. (2020). Experimental demonstration of underwater ultrasound cloaking based on metagrating. Appl. Phys. Lett..

[150] Xu Z., Wang Y., Wu D., Ananth K.P., Bai J. (2019). The process and performance comparison of polyamide 12 manufactured by multi jet fusion and selective laser sintering. J. Manuf. Process..

[151] Roca D., Pamies T., Cante J., Valls O.L., Oliver J. (2020). Experimental and numerical assessment of local resonance phenomena in 3D-printed acoustic metamaterials. J. Vib. Acoust..

[152] Wang P., Li X., Jiang Y., Nai M.L.S., Ding J., Wei J. (2020). Electron beam melted heterogeneously porous microlattices for metallic bone applications: Design and investigations of boundary and edge effects. Addit. Manuf..

[153] Novak N., Krstulović-Opara L., Ren Z., Vesenjak M. (2020). Mechanical properties of hybrid metamaterial with auxetic chiral cellular structure and silicon filler. Compos. Struct..

[154] Yang H., Yang S., Chi X., Evans J.R.G. (2006). Fine ceramic lattices prepared by extrusion freeforming. J. Biomed. Mater. Res. Part B Appl. Biomater..

[155] Hirt L., Reiser A., Spolenak R., Zambelli T. (2017). Additive manufacturing of metal structures at the micrometer scale. Adv. Mater..

[156] Udofia E.N., Zhou W. Microextrusion based 3D printing—A review. Proceedings of the 29th Annual International Solid Freeform Fabrication Symposium—An Additive Manufacturing Conference.

[157] Abeykoon C., Sri-Amphorn P., Fernando A. (2020). Optimization of fused deposition modeling parameters for improved PLA and ABS 3D printed structures. Int. J. Lightweight Mater. Manuf..

[158] Vaezi M., Sejo Z., Humphrey V., Yang S. Feasibility study of printing acoustic metamaterials using extrsuion freeforming method. Proceedings of the 1st International Conference on Progress in Additive Manufacturing.

[159] Postiglione G., Natale G., Griffini G., Levi M., Turri S. (2015). Conductive 3D microstructures by direct 3D printing of polymer/carbon nanotube nanocomposites via liquid deposition modeling. Compos. Part A Appl. Sci. Manuf..

[160] Jiang P., Ji Z., Zhang X., Liu Z., Wang X. (2017). Recent advances in direct ink writing of electronic components and functional devices. Prog. Addit. Manuf..

[161] Gratson G.M., García-Santamaría F., Lousse V., Xu M., Fan S., Lewis J.A., Braun P.V. (2006). Direct-write assembly of three-dimensional photonic crystals: Conversion of polymer scaffolds to silicon hollow-woodpile structures. Adv. Mater..

[162] Kolesky D.B., Homan K.A., Skylar-Scott M.A., Lewis J.A. (2016). Three-dimensional bioprinting of thick vascularized tissues. Proc. Natl. Acad. Sci. USA.

[163] Vdovin R., Tomilina T., Smelov V., Laktionova M. (2017). Implementation of the additive PolyJet technology to the development and fabricating the samples of the acoustic metamaterials. Procedia Eng..

[164] Liashenko I., Rosell-Llompart J., Cabot A. (2020). Ultrafast 3D printing with submicrometer features using electrostatic jet deflection. Nat. Commun..

[165] Peng X., Kuang X., Roach D.J., Wang Y., Hamel C.M., Lu C., Qi H.J. (2021). Integrating digital light processing with direct ink writing for hybrid 3D printing of functional structures and devices. Addit. Manuf..

[166] Yang Y., Li X., Zheng X., Chen Z., Zhou Q., Chen Y. (2018). 3D-printed biomimetic super-hydrophobic structure for microdroplet manipulation and oil/water separation. Adv. Mater..

[167] Laumer T., Stichel T., Amend P., Schmidt M. (2015). Simultaneous laser beam melting of multimaterial polymer parts. J. Laser Appl..

[168] Zhao L., Laredo E., Ryan O., Yazdkhasti A., Kim H.-T., Ganye R., Horiuchi T., Yu M. (2020). Ultrasound beam steering with flattened acoustic metamaterial luneburg lens. Appl. Phys. Lett..

[169] Kennedy J., Flanagan L., Dowling L., Bennett G.J., Rice H., Trimble D. (2019). The influence of additive manufacturing processes on the performance of a periodic acoustic metamaterial. Int. J. Polym. Sci..

